# Endophytic and Rhizospheric Microorganisms: An Alternative for Sustainable, Organic, and Regenerative Bioinput Formulations for Modern Agriculture

**DOI:** 10.3390/microorganisms13040813

**Published:** 2025-04-03

**Authors:** Isabela de L. Valente, João H. C. Wancura, Giovani L. Zabot, Marcio A. Mazutti

**Affiliations:** 1 Department of Chemical Engineering, Federal University of Santa Maria (UFSM), 1000 Roraima Av., Camobi, Santa Maria 97105-340, RS, Brazil; isabelavalentee@gmail.com (I.d.L.V.); marciomazutti@gmail.com (M.A.M.); 2 Laboratory of Biomass and Biofuels (L2B), Federal University of Santa Maria (UFSM), 1000 Roraima Av., Camobi, Santa Maria 97105-340, RS, Brazil; jhwancura@hotmail.com; 3 Laboratory of Agroindustrial Process Engineering (LAPE), Federal University of Santa Maria (UFSM), 3013 Taufik Germano Rd, Universitário II, Cachoeira do Sul 96503-205, RS, Brazil

**Keywords:** sustainable bioinputs, biological inputs, phytohormones, biocontrol, symbiosis

## Abstract

Large amounts of chemical fertilizers are still used to suppress pathogens and boost agricultural productivity and food generation. However, their use can cause harmful environmental imbalance. Furthermore, plants typically absorb limited amounts of the nutrients provided by chemical fertilizers. Recent studies are recommending the use of microbiota present in the soil in different formulations, considering that several microorganisms are found in nature in association with plants in a symbiotic, antagonistic, or synergistic way. This ecological alternative is positive because no undesirable significant alterations occur in the environment while stimulating plant nutrition development and protection against damage caused by control pathogens. Therefore, this review presents a comprehensive discussion regarding endophytic and rhizospheric microorganisms and their interaction with plants, including signaling and bio-control processes concerning the plant’s defense against pathogenic spread. A discussion is provided about the importance of these bioinputs as a microbial resource that promotes plant development and their sustainable protection methods aiming to increase resilience in the agricultural system. In modern agriculture, the manipulation of bioinputs through Rhizobium contributes to reducing the effects of greenhouse gases by managing nitrogen runoff and decreasing nitrous oxide. Additionally, mycorrhizal fungi extend their root systems, providing plants with greater access to water and nutrients.

## 1. Introduction

Plants have an essential importance in the ecosystem with the mastery of sustaining the system and fixing energy in the biosphere [1]. However, in a natural environment, crops can be constantly affected by pathogens with the consequent proliferation of diseases, reducing agricultural production, and threatening food security [2]. The phytopathogenic organisms that cause damage to crops include bacteria, fungi, viruses, nematodes, oomycetes, and insects [3]. Furthermore, the crops may be damaged by other poorly managed vegetation [1]. Therefore, it is crucial to control diseases to minimize losses in agricultural production.

Despite suffering from biotic and abiotic agents that promote stress, plants typically have a natural defense system against these attacks [4], such as the Acquired Systemic Acclimatization, triggered in the presence of abiotic causes [5], in addition to physical and biochemical barriers to biotics [6,7]. Vegetables have induced defenses in parts of the plant that have not yet been exposed to harm [5]. They have specific immunological responses to each invasion attempt, whether in their external or internal layers for both insects or microorganisms, providing defense reactions to non-host components or hypersensitivities to induced resistance [6,7].

Another concern extends to population growth and, consequently, to an increase in demand for food production. Land use is increasing, and more chemical fertilizers are applied. In this way, the soil is being eroded, affecting its microbiota. The chemical products being used to control the plants’ diseases have a short-term duration, causing damage to the environment and human health and the emergence of resistant genotypes [8]. Considering the concept of the bioeconomy, efforts have been made to improve and balance food production as well as soil management through the use of bioinputs, such as biostimulants and bioinoculants [9]. Bioinoculants and biofertilizers belonging to the genera *Aspergillus*, *Rhizoctonia*, *Penicillium*, *Piriformospora*, *Phoma*, and *Trichoderma* are interesting alternatives to phytochemicals, having notable resources in controlling diseases and enhancing the productivity and quality of crops [10].

A favorable collaboration between plants and microorganisms exists, providing better development and overcoming the plant system against biotic and abiotic stresses by interfering with the immune system, designating a bilateral relationship between them, such as endophytic microorganisms [1]. As an example of an endophytic microorganism, the fungus *Trichoderma* spp. (*T. harzianum*, *T. asperellum*, and *T. viride*) is frequently used in biocontrols and the biosynthesis of industrial enzymes (chitinases, glucanases, and proteases) [11,12]. Therefore, these microorganisms demonstrate that they are capable of being used both in plant defense against biotic and abiotic stress and as bioinoculants that contribute to plant development [12]. Furthermore, rhizobacteria share applicability with root endophytic bacteria, where endophytic and mycorrhizal fungi increase phosphate solubilization in addition to the production of siderophores and organic acids that contribute to the movement of minerals from the soil to the leaves [13].

Considering the exposed arguments, this paper presents a comprehensive discussion regarding the relationship between plant versus endophytic and rhizospheric microorganisms, including signaling processes, barriers, and plant defense in the presence of pathogens. This review describes the potential of bioinputs as an alternative to control pests and provide nutrients. Bioinputs promote the growth and development of plants, focusing on the use of endophytic microorganisms as part of the plant microbiota or the surrounding soil microorganisms.

## 2. Methodology of Review

This review aims to perform a meta-analysis on the application of soil microbiota as a microbial resource through bioinputs, presenting a sustainable alternative to the agricultural system. Given the extensive heterogeneity, systematic research and analysis of scientific articles, and occasionally patents, were conducted using keywords such as “bioinputs”, “endophytic microorganisms and rhizospheric”, “soil microbiota”, “phytohormone”, and “plant defense system”. Following the selection and evaluation of eligible documents, with a focus on predominantly recent approaches, the compiled data were strategically organized for better understanding. The search began in October 2021 and was conducted until December 2024 through platforms such as ScienceDirect, Scopus, Capes Journals, and Espacenet.

## 3. Endophytic Microorganisms

Endophytic microorganisms reside in plants during all or part of their life cycle in a determined habitat without causing visible or internal harm (diseases or symptoms) [14,15]. The word “endophyte” is the combination of two Greek words: “endon” meaning “from within or internal” and “phyton” which means “plant” [12]. When described as “obligate endophytic” microorganisms, we are dealing with the relationship between the time of permanence of the endophytic component in the host and when they complete their entire life cycle. Another particularity refers to that they can be facultative, where the host selects the microorganisms or the host can be used as a dissemination vector. Anyway, there is a consumption of nutrients and a decrease in the plant’s predisposition [16].

Endophytic microorganisms that develop in external parts attached to plant tissues (epiphytes) are named opportunists and can eventually move to the internal part of the plant (endosphere) [8]. Some inherent classifications present the location in which microorganisms are found. When they reside on the surface above the ground of plants, they are called phyllosphere, also presenting a specific subdivision where the microbiota is essentially located. Examples of surfaces are stems, flowers, fruits, and leaves, which are called caulosphere, astosphere, carposphere, and phylloplant, respectively. Similarly, another region of the plant that provides more nutrients and less microbial diversification compared to the phyllosphere is the roots, called the rhizospheric region. The third region is named endosphere, corresponding to the entry of microorganisms into the plant’s internal tissues, subdivided into endorrizosphere (when residing in the root) and endophyllosphere (covering the internal stem, leaf tissue, reproductive tissue internal, and the seeds) [17].

Phyllospheric microorganisms enable the development and resistance of plants, in addition to the biosynthesis of metabolites and phytohormones, nitrogen fixation, and safeguarding against phytopathogens. As an example of striking phytobiomes in some plant species, there are bacteria such as *Pseudomonas*, *Bacillus*, and *Pantoea* [8,16]. Nevertheless, they enable plants to adapt against abiotic stresses [17], in correlation with secondary metabolites, in the production of antifreeze proteins, antioxidants, and proline and polyamine [18]. Endospheric microorganisms are characteristic of plant growth through modulation of metabolic correlations, amplification of nutrient absorption, and increased tolerance to biotic and abiotic stresses [17].

Microbial assistance in the development of plants, such as rhizobacteria and some fungi, is of vital relevance in agriculture when their nutritional supply is scarce [18]. Rhizospheric microorganisms may be beneficial to the host, but they provide essential nutrients such as phosphorus and nitrogen as well as assistance in photosynthesis (mycorrhizal fungi and rhizobia), and the supply of iron (siderophore bacteria) [16,17]. In addition, they can promote the growth of plants (rhizobacteria) and stop diseases linked to phytopathogenesis. These microorganisms adjust the production of plant hormones (auxin (AUX), cytokines (CKs), gibberellin (GAs), ethylene (ET), and abscisic acid (ABA)), which are mediators of plant growth and root assimilation, promoting increased nutrient absorption [18].

Phytobiome communities can be abundant and may modify their composition at intra- and interspecific levels in plant hosts, according to environmental conditions and plant development [17]. Endophytic microbial biocenosis is affected by several circumstances regarding the species and genotype of the host and the association between them, such as the type of plant tissue, habitat, lack of nutrients, and abiotic and biotic stresses [19].

Endophytic microorganisms can activate the plant’s defense response against phytopathogens, a phenomenon known as induced systemic resistance (ISR). Evidence suggests that the initial contact between the microorganism and the plant, even when beneficial, triggers a response similar to the plant’s immune reaction against pathogens. However, through mutualistic adaptation, these microorganisms can circumvent the defense response and establish colonization without significant issues. Key bacterial factors involved in inducing systemic resistance include flagella, antibiotics, homoserine N-acyllactones, salicylic acid (SA), jasmonic acid (JA), siderophores, volatile compounds (such as acetoin), and lipopolysaccharides [18].

The factors provided by fungi, which are more related to the ability to produce compounds with inhibitory activity on the growth of phytopathogens and herbivores, are alkaloids, steroids, terpenoids, peptides, polyketones, flavonoids, quinones, phenols, and chlorinated compounds [16,18]. Therefore, the noticeable manifestation of diseases in plants corresponds to the response of plant physiology to the association between plants and microorganisms, which sometimes may not be easy to identify in terms of symbiotic, antagonistic, or synergistic relationships [20]. Consequently, the difference between endophytic, epiphytic, and phytopathogenic organisms is associated with the instructive condition of each one [21].

## 4. Plant Defense Mechanism

Plants have their defense systems against adverse situations and, consequently, against microorganisms through multilayers with inducible responses. Phytopathogenic microorganisms are distinguished as biotrophic, where they acquire nutrients through living cells, commonly do not generate toxins, and partially expel enzymes. As examples, there are the omycetes (*Hyaloperonospora arabidopsidis*) and the hemibiotrophs, which extract nutrients from the host cells that slaughter, having biotrophic and necrotrophic traces such as bacteria (*Pseudomonas syringae*); or necrotrophic organisms, which obtain nutrients from dead cells and secrete toxins and enzymes that deteriorate the cell walls of plants such as fungi (*Botrytis cinerea*) [22]. The main factor that differentiates them is the mode of infection to plants and the feeding system, which can reduce or decimate biomass, affecting the quantity and quality of agricultural manufacturing [23].

Typically, protective mechanisms resist infection attempts through physical and biochemical barriers. Most plant species acquire a form of resistance known as non-host resistance characterized by its long duration compared to R genes. These barriers are constitutive defenses (such as the cuticle and cell wall) and inducible, corresponding to the accumulation of secondary metabolites synthesized after infection [6]. Once this obstacle is overcome, it is observed that the hypersensitivity reaction generates necrosis in the infected area [7].

### 4.1. Physical Barrier

#### 4.1.1. Cuticle

The plant’s defense systems that cause non-host resistance are the size of the cell wall (cerumen on the outside of the cuticle), the actin protein filaments, the production of glucosinolate metabolites, the chemical compound phytoalexins, and the creation of lignin, and the incitement of proteins belonging to pathogenesis (conferred to microbial presence) [24]. The aerial fractions of plants (leaves, fruit, flowers, seeds, and non-woody stems) have a hydrophobic coating. In addition to controlling non-stomatal water loss, the spread of molecules, and gas exchange, the coating establishes the first interaction with the environment and also the first stage of defense against pathogens, which is a characteristic equivalent to its physical barrier [25]. This protective layer consists of the plant’s cuticle, which diversifies according to its species and ontogeny [26].

The plant cuticle is composed of a lipophilic coating corresponding to cuticular wax integrated with a layer of cutin, biosynthesized from fatty acids. The cutin is made up of C_16_ and C_18_ molecules, interesterified in each other and with other substances such as glycerol, phenylpropanoids, and dicarboxylic acids [26] that have undergone hydroxylation and epoxylation modifications generating cutin monomers, such as ω-hydroxyacids, polyhydroxyacids, epoxy, and α-/ω-dicarboxylic acids [27,28].

**Figure 1:**
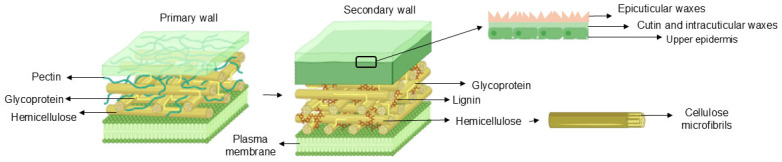
Representation of the subcellular location of vegetable epicuticular waxes, cutin, intracuticular waxes, cell wall, its ordered complex (primary and secondary cell wall, in addition to the cellulose microfibril), and the plasma membrane.

Similarly, cuticular wax consists of a combination of long-chain fatty acids, alkanes, primary and secondary alcohols, esters, ketones, and aldehydes [26,29], in addition to different proportions of triterpenoids and phenylpropanoids [29]. It is usual for certain species to present determined secondary metabolites such as phenols, flavonoids, tocopherols, and cyanamic acids [30]. Furthermore, cutillary wax has two segments according to its position in the cuticle (intracuticular and epicuticular). Above the cutin, there is the epicuticular wax (responsible for the resplendence of leaves and fruits) and closer to the cell wall there is the intracuticular wax [31] (Figure 1).

Although the conjunction between the cuticle and the flat cell wall is not sufficient in protecting against fungi, its importance in shielding against the penetration of bacteria is well-known, although other natural gaps may allow this invasion. In this scenario, bacteria can use strategies such as hydathodes, nectardes, lenticels, or stomata. Notwithstanding, wounds promoted by herbivores and climatological phenomena such as winds and storms can establish further infection mechanisms [23].

#### 4.1.2. Cell Wall

The cell wall consists of an ordered complex of biopolymers formed by the elementary triad “cellulose—hemicellulose—lignin”, in addition to glucans, glycoproteins, and water [32]. This complex is responsible for plant growth and morphology [33], establishing mechanical properties in tissues and regulating adhesion between cells [32]. Although depending on biotic or abiotic stress, its modification may occur according to momentary needs [15].

As represented in Figure 1, during plant development, its cell wall is divided into a primary layer and a secondary layer. The growing layer is the primary layer, consisting mainly of cellulose, hemicellulose, and pectin [15,34]. The primary layer is further subdivided into type I and type II. Although they present equivalent amounts of cellulose, there are typically differences in the amount of hemicellulose, found more pronounced in the type II primary layer [35]. The type I-primary layer has a considerable amount of pectin that can supply its smaller amount of hemicellulose [32]. Thus, there is an intertwining of hemicellulose with cellulose microfibrils, forming a network that is introduced into a matrix composed of pectin and hemicellulose [36]. At the end of plant expansion, the plant typically needs structural reinforcement from its secondary layer composed of cellulose, hemicellulose, and lignin, which has higher rigidity and recalcitrance [15,34]. The secondary layer also has a tangle of semi-crystalline cellulose microfibrils inserted in an amorphous matrix composed of hemicellulose and lignin, where its thickness can vary according to physicochemical properties [35].

As protection against stress or injuries, or during the plant’s growth phase, lipid sedimentation often occurs. This phenomenon comes from ester-linked fatty acids containing glycerol molecules in their structure in higher degrees of hydroxycinnamic acids (ferulate in elevated concentrations, fatty alcohols, aliphatic saturates compounds, and molecules with 20 carbons) within the primary cell wall, corresponding to a barrier named suberin (Figure 1). The function consists of mineral nutrition and, similarly to the cuticle, it is a water regulator and promotes resistance to pathogens [27]. Furthermore, the cell wall safeguards plant cells from invaders. However, endophytic microorganisms can produce enzymes (cellulase, lactase, pectinase, and xylanases) to assist in the absorption of nutrients at the same time that cause the penetration of pathogens into the plant and their colonization and proliferation [15].

#### 4.1.3. Cytoskeleton

The plant cytoskeleton represents a grouping of fibers found in the plant cytoplasm, comprising microtubules (tubulin) and microfilaments (actin), responsible for intracellular transport [37]. Endomembranes, which are surrounded by proteins that undergo successive structural modifications, also support the passage of biosynthetic loads, helping in the maturation of cells and supplying defense molecules [38]. Consequently, actin filaments are involved in the strict relationship between plants and microorganisms. Such filaments consist of a dynamic restructuring of actin-binding proteins, actin-binding proteins profilin (cyclase-associated protein or adenylate cyclase-associated protein), actin depolymerization factor, and Rac (Rho-GTPase). The alteration in the gene that encodes the actin depolymerization factor promotes the inactivation of non-host resistance [37], where one of the defense pathways that triggers pattern-triggered immunity (PTI) consists of a degree of actin concentration in the infected region [38].

### 4.2. Biochemical Barrier

After overcoming physical barriers, the invading microorganism needs to pass through other protective resources produced by plants. Such resources include antimicrobial compounds (phytoanticipins) that prevent the action of phytopathogens, as well as secondary metabolites, consisting of the plant’s biochemical barrier [39,40]. These metabolites are designed in the plant as responses to biotic and abiotic stress scenarios [41].

Glucosinolates stand out among the main metabolites developed by plants. Chemically, these compounds are categorized as β-thioglucoside-N-hydroxysulfates, having sulfur and nitrogen in their structure. Glucosinolates are typically related to the *Brassicaceae* family and are also present in *Capparaceae* and *Resedaceae* species, in addition to plants from *Euphorbiaceae*, *Tovariaceae*, *Moringaceae*, *Tropaeolaceae*, and *Caricaceae* [42]. These metabolites are constantly generated by plants even without the presence of pathogens. They are part of the group of molecules known as phytoanticipins, although some glucosinolates resulting from tryptophage can act as phytoalexins.

Despite having limited biological activity, glucosinolates undergo structural modifications in the presence of infection, thus being used as induced immunity [43]. As a result, these metabolites may present inactive precursors, accumulating at the infection local (or being transported by the xylem or phloem—or even through enzymes—to more distant locations) [44,45]. In addition, these plants have myrosinases, responsible for the glucosinolates hydrolysis, which results in epithionitriles, thiocyanates, nitriles, and isothiocyanates that are recognized for being leaders of distinct biological activities [42].

Terpenoids, flavonoids, and alkaloids are phytoalexins biosynthesized by several pathways, induced in the presence of pathogens, ultraviolet irradiation, and elicitor interventions (N-acetylcytooligassarides, jasmonium acid, and CuCl_2_) [39,40]. However, the molecular patterns associated with microorganisms or pathogens stimulate phytoalexins at the local of infection, through the activation of mitogen-activated protein kinases (MAPKs), which increase their signal, promoting gene transcription and metabolite accumulation, limiting pathogen proliferation [44].

Usually, phytoalexins are also stimulated by hypersensitivity reactions. The living cells surrounding the eradicated cells are required to produce the antimicrobial compounds, making the cells “more alert” to future infections. Although the hypersensitivity reaction occurs quickly, the accumulation of phytoalexins occurs more slowly [46]. The protection mechanism of plants is specific for each microorganism, such as the use of the type III secretion system by bacterial pathogens and the haustoria by fungi [38]. Thus, the cell death of the pathogen is driven at an early stage as soon as it is accessed, with no possibility of dissipation around the lesion or inside the cells [25].

## 5. Molecular Patterns

### 5.1. Elicitors

The immune system is being modified (or co-evolving) over time, thus fitting in the “Red Queen hypothesis”. This hypothesis considers that both the host and the pathogen adapt and can have defense reactions, resisting the immunization actions (virulence), as a type of “race in place”, as described by the biologist Leigh Van Valen in 1973 [47]. Pathogens prefer common genotypes to rare ones in plants, characterizing the phenomenon as a negative frequency-dependent selection, which can occur with a variety of ecological and epidemiological factors [20]. In a specified area, local plants may be affected by pathogens, but not necessarily the entire population of the species in that region.

**Figure 2:**
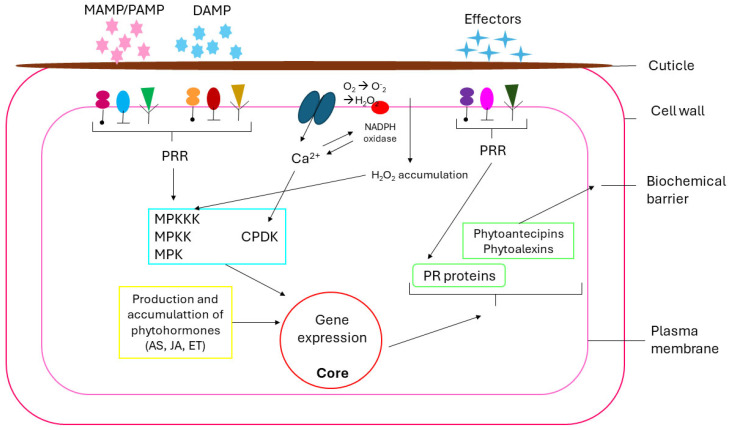
General and specific elicitors of plants.

In natural floras, there are often resistance genes that exert relevant functions (pondering to habitual) in their immunological reactions. The dominant and occasionally recessive genes provide total or partial resistance to one or more pathogens, caused by the selection and adaptation of certain plants. Pathogen-associated molecular patterns (PAMPs) and microorganism-associated molecular patterns (MAMPs) compose the resistance genes with the same resistance characteristic, which come from non-pathogenic microorganisms, thus being able to use both forms to reference molecular patterns [48], according to represented in Figure 2. Also, there are damage-associated molecular patterns (DAMPs), which are classified as elicitors [20].

Elicitors are responsible for inducing plant defense responses, both in leaves and roots through non-pathogenic or pathogenic microorganisms (PAMPs/MAMPs) [2,49]. In this process, compounds are released from plant cell walls through biotic or abiotic stresses and even when there is production or secretion in vegetables when damage occurs to their cells (DAMPs) [50]. Microbiotas in the condition of pathogenesis are usually linked to bacterial flagella, peptidoglycan, fungal chitin, and the elongation factor Tu [2,38,49].

Elicitors are compounds released from the cell walls of pathogenic or non-pathogenic organisms, plant secretions, or abiotic stresses, recognized by pattern receptors. These receptors also recognize effectors, molecules with virulent factors. From this, signaling cascades are released to induce defense responses, such as PTI (Pattern Triggered Immunity) [48], and lead to non-host resistance, for the elicitors. Through recognition by the resistance gene that encodes the resistance protein inciting effector-triggered susceptibility, the effector-triggered immunity corresponding to hypersensitivity reaction responses is triggered [20,48].

Elicitors are also recognized by the transmembrane pattern receptors or pattern recognition receptors (PRRs) present in plasma membranes and incorporated by endocytosis [38]. In its structure, there are also leucine-rich extracellular repeats (LRRs) in addition to intracellular kinase domains with a configuration of heteromeric surface receptor compounds [49]. Thus, two receptors (receptor-like kinases—RLKs and receptor-like protein—RLPs) act as PRRs, where the RLKs have a transmembrane domain with extracellular ligand uptake and an intracellular kinase domain, with the major difference between RLKs and RLPs is that the second does not present the kinase domain [20].

In addition to LRRs, PRRs have the lysin motif (LysM), lectin, and epidermal growth factor (EGF) domains, providing interesting coverage in their recognition. Consequently, PRRs form a cluster with co-receptors that have equivalent domains such as receptor-like cytoplasmic kinases (RLCKs). Thus, when activated, they stimulate plant immunization procedures, as this tangle stimulates the calcium-dependent protein kinases and MAPKs, which transmit defense signals [20]. The defense signals involve the immunity triggered by molecular patterns associated with innate pathogens or pattern-triggered immunity and/or microorganisms-triggered immunity (MTI) [51], which protect against non-host pathogens, not instituting virulence while leading to higher resistance, such as NHRs [6].

Protein kinases are essential segments in this process, signaling translation proteins, where MAPKs intensify this signal through interconnections [46]. The cascades of the protein kinase are composed of three protein kinases that operate by phosphorylation, such as MAPK, MAPK-kinase (MAPKK), and MAPK-kinase-kinase (MAPKKK). Thus, the MAPKKKs activate the MAPKKs by phosphorylation of serine and threonine residues and subsequently, the MAPKKs activate the MAPKs, also by phosphorylation, nonetheless by threonine and tyrosine residues. Subsequently, stimulated MAPKs are capable of driving the expression of genes involved, operating in effector proteins as well as transcription factors [52].

The intracellular Ca^2+^ can have its concentration modified and transition promoted by stimuli such as light, hormones, and biotic and abiotic stresses. Therefore, specific sensors are responsible for detecting, decoding, and translating them, just like the phosphorylation of the target protein. This translation occurs through the synergistic reaction between calmodulin (CaM) and the CDPKs. An example of ion flux equivalent to Ca^2+^ concentration, where its intracellular increase stimulates the β-1,3-glucan, is the synthase responsible for converting UDP-glucose into β-1,3-glucan polymers, succeeding in a subsequent accumulation of callus in the plasma membrane attached to the cell wall. It slows pathogenic spread while the plant mounts transcriptional defense responses [53].

Other corresponding responses of the elicitors are the triggering of signaling cascades, such as ion flow, oxidative explosion reaction (reactive oxygen species—ROS—superoxide and hydrogen peroxide), and synthesis of AJ and AS as secondary messengers. When inducing microbial intervention genes, changes typically occur in plant mRNA, resulting in the production of transcription-encoding implicit defense proteins [54]. H_2_O_2_ e O_2_**^•^**^−^ are drivers of both plant defense and development in response to abiotic stresses, while cellular groups such as mitochondria, chloroplasts, and peroxisomes are sources of ROS to biotic and abiotic stresses.

Consequently, the production of ROS by the plant organism consists of a common effect as a by-product of cellular metabolism, while at the same time, they can be harmful, such as hydrogen peroxide (H_2_O_2_), superoxide anion (O_2_^•−^), singlet oxygen (^1^O_2_), and hydroxyl radical (•OH). Furthermore, these free radicals can function in signaling pathways, where it is important to describe that their accumulation provides their responsibilities in the cell. Even at lower levels, they can act as translation molecules. At higher levels, they can be toxic molecules with oxidizing capacity. Thus, the plant defense system has two response stages in the presence of microorganisms, known as oxidative explosion. The first occurs at a transient production of reduced amplitude through MAMPs/PAMPs, followed by the second stage with higher accumulation of ROS due to the hypersensitivity reaction, causing cell death [55].

The ROS produced inside of the infected cells are responsible for cell wall lignification, functioning as a method of defense against microbial penetration by activating vegetable resistance mechanisms. At high levels, the reactions provide the biosynthesis of phytoalexins and the cross-linking of cell wall glycoproteins with excessive proline. However, plants can also produce antioxidant enzymes that disable cell-damaging radicals, such as catalases, ascorbate peroxidase, peroxiredoxins, glutathione/thioredoxin, peroxidases, and glutathione S-transferase [46].

### 5.2. Effectors

Inside the plant cells, another level of perception occurs due to the continuous adaptation of pathogens to vegetable species, where microorganisms secrete effector molecules with virulent factors in the apoplast or cytoplasm, covering the cytoskeleton and plant organelles [38]. Such molecules, when detected by the R-gene, initiate a signaling cascade that intervenes in the microbial PTI response, either evading or nullifying the perceptions of the PAMPs/MAMPs by exciting an immune response by effector-triggered susceptibility (ETS) [49]. More precisely, the R-genes encode the R-protein that is the intracellular receptor with nucleotide binding and leucine-rich domains (NLRs—nod-like receptors), occurring through the binding of an ATPase binding domain (NB) and another rich in leucine (LRR), which incites the ETS [49,56].

There are several types of R-genes and their prevalence is polymorphic, a characteristic attributed to plant breeding programs. However, the vast majority of R-genes are analogous, not providing resistance to plants, although they can assist, when linked to certain species, in the resistance of the non-host organism. Several R genes recognize pathogen effectors, that is, the activation of specific NLRs (or their pairs). Through the action of one or more effectors, they trigger ETI (Effector-Triggered Immunity), corresponding to hypersensitivity reaction responses such as cell death programming, usually demanding defense against biotrophic takeovers that possess living cells during their proliferation [50]. The PAMP (PTI) differs from ETI because it does not involve hypersensitivity reactions [48].

After the pathogen identification, plants trigger defense responses, which also include the closure of stomata, the reduction of nutrients from the cytosol to the apoplast (hindering bacterial multiplication), the phytoalexins production (such as camalexins), and ROS generation [23]. The ROS, reactive nitrogen species (RNS), and nitric oxide (NO), can modulate the HR [57]. Thus, during the incitement of hypersensitivity reaction, changes in plant metabolism may occur, such as lignification, suberization, callose deposition, changes in ion pathways, and lipid peroxidation. In programmed cell death, there is an oxidative explosion with the formation of superoxide, hydrogen peroxide, and concentration of hydroxyl radical [55].

The cytoskeleton is related to different PRRs where, when activated, they are internalized by endocytosis, being coated by a clathrin protein vesicle. The process occurs with the displacement of these elements to the early endosome (EE). The trans-Golgi network (TGN/EE), when distributing the receptor, recycles the receptors, and keeps the collected material in the endosomal system, with the late endosome sent for degradation in vacuoles. These effectors can impede transaction processes, leading to the formation of calluses in plant plasmodesmata, making it impossible to connect between cells [38].

The NB-LRRs (NLRs) proteins have two types of classification, depending on the existence of a TIR domain (interleukin-1 receptor). The domain is denominated TIR-NB-LRR when its presentation occurs at the N-terminus (TNLs) and nTIR-NB-LRR (or CC-NB-LRR) when this aspect is not verified (CNLs) [21]. Furthermore, NLRs recognize pathogenic effectors in obligate biotrophic or hemibiotrophic hosts, activating the plant defense system. This recognition is not verified for necrotrophic hosts, which kill plant tissue, where the difference in the defense system to distinguish these hosts consists of the involvement of AJ and ET. In this arrangement, the identification by NLRs promotes an accumulation of AS and ROS at the site of infection, characteristic of distinct biotrophic hosts [57]. As for ROS, some biotrophic pathogens are prevented by the action of H_2_O_2_, benefiting necrotrophic organisms until the radicalization of this compound is verified [55].

In this panorama, ETI boosts mobile immunological manifestations in plants, such as methyl salicylic acid (MeSA), azelaic acid, and glycerol-3-phosphate (G3P), which are displaced from the site of infection to non-infected tissues. In these tissues, where contamination does not occur, there is a concentration of MeSA and the stimulation of genes encoding pathogenesis-related proteins (PRs) that protect the plant from subsequent attacks. PR proteins are a set of structurally dissimilar plant proteins (or pathogen-associated enzymes) engaged in HP responses that promote ROS incitement, cell wall strengthening, antibiotic synthesis, and programmed cell death [58].

The induction of immunological defense is known as systemic acquired resistance (SAR), providing higher challenge/resistance to phytopathogens, with its incitement accomplished through antibiosis to the phytopathogen, promoting necrosis [51]. According to Ghosh et al. [46], SAR does not eradicate pathogens, acting only on the mitigation and reduction of infections, persevering for a long period in the plant structure as a “working memory” that can be transferred to subsequent generations through “transgenerational immunity” by epigenetic systems (DNA methylation and chromatin modification). The long-term defense occurs through the accumulation of several procedures (ROS, PTI, endogenous release of AS, expression of PR, and HR).

According to Fernandez-Calvo et al. [59], the activation of PTI in plants through glycans derived from the plant cell wall (DAMPs) and extracellular pathogen-derived molecules (MAMPs) constitutes a substantial system for an efficient disease resistance response during plant-microorganism interactions. Thus, *Arabidopsis thaliana* recognizes, through certain PRRs such as LysM RKs, the β-1,4-D-XYL4 oligosaccharides and β-1,6-D-Glc branchings, which trigger PTI.

Li et al. [60] predicted 67 effector proteins, which were expressed in *Nicotiana benthamiana* leaves to identify which would induce cell death through Bcl-2-associated X (BAX). The effector VdCE51 was identified, which also suppressed cell death induced by INF1 (a PAMP from *Phytophthora infestans*), VdNLP1, and VdCE11 (a cell death inducer and an effector of *Verticillium dahliae*, respectively). This process occurred when BAX induced the expression of NbH1N1 and NbHSR203J (specific marker genes of HR), which were subsequently suppressed in the co-expression of BAX with VdCE51, generating HR-associated immunity.

The phytopathogen *Fusarium sacchari* possesses Nis1 as the structural domain of the effector FsNis1, a virulence factor that induced cell necrosis in *N. benthamiana* and sugarcane. The effectors BAX (positive control), FsNis1, and FsNis1ΔSP, when inoculated in *N. benthamiana*, exhibited sites with ROS bursts. Regarding the presence of callose and comparing BAX, FsNis1, FsNis1ΔSP, and EV (empty vector—negative control), BAX showed the highest callose accumulation [61].

## 6. Plant Growth Promoters

### 6.1. Phytohormones

The acquisition of plant immunity consists of a well-organized complex network. Hormones are part of this process, playing a significant role in regulating immune responses [62]. Thus, phytohormones are small signaling molecules that help in the growth and development of plants. Plants that have genes that express SAR have higher systemic quantities of some hormones (AS, AJ, and ET, as represented in Figure 2), responsible for signaling that incites defense responses and transcriptional reprogramming of vegetables [18]. Phytohormones AS and AJ refer to the primary signs of immune reactions, and can be triggered as hormonal treatment or as molecules and metabolites in the presence of microorganisms. In the detection of MAMPs/PAMPs and the consequent slow defense reaction, such as kinase cascades and ROS production, the expression of genes and accumulations of defense hormones (similar pato PTI) can trigger SAR with PR agglomeration in adjacent locations [57,63]. Likewise, prompting ISR may occur with an abundance of specific proteins, phytoalexins, and an increase in callus through AS for SAR or dependent on ET/AJ in plants for ISR [64].

After recognition by PAMPs/MAMPs, AS biosynthesis occurs during the PTI, as well as the ETI through effectors. This production occurs through a microbial transition that induces the flow of Ca^2+^, signaling the AS production [1]. The AS, in addition to being a growth agent with a phenolic nature in physiological alignment with plants, also hinders the activity of catalases, promoting elongation in H_2_O_2_ levels while providing stimulation of the PR protein [65].

Jasmonates (AJs), and their derivatives are obtained from lipids and belong to the oxylipin family, characteristic of the α-linolenic acid oxidation [66]. Furthermore, Hajrah et al. [66] describe that the combination of AJ and ET in *Catharanthus roseus* provided the production of phenolic compounds, such as AS, benzoic acid, and cyanamic acid. ET is a gaseous vegetable hormone that contributes to several parameters in plant growth. These hormones are responsible for the ripening of fruits, also acting as a signal for the development of infections, as in the case of *Colletotrichum gloeosporioides* and the intervention of *Botrytis cinerea* germination [67].

ABA is responsible for balancing factors in the plant’s physiology, its progressions, dormancy, seed twinning, and environmental stress. It is capable of inciting defense reactions such as stomatal closure, enzymes with antioxidant activities, water control, and stimulation of transcription in reduced times [68]. ABA is significant in responses to stress as it increases the concentration of osmolytes (glycine, betaine, and proline) in the plant to maintain its osmotic balance [69].

ET contributes to the ripening of fruits and seed germination, and like ABA, it is stress phytohormones, inhibiting vegetable development. In this panorama, studies on the interaction between ET and ABA report that the inhibition of ET causes an increase in ABA (example: rice seedlings) ET compensates for ABA deficiency (example: maize mutants), in which higher ET production can promote ABA accumulation (example: Arabidopsis mutant) [70]. According to Sharma et al. [71], GAs are related to other plant processes that include seed germination, sexual expression, fruit formation, stem extension, and senescence.

AUXs are related to the development of the cell cycle, where exogenous auxin inhibits the signaling of defense reactions inherent to AS, activating resistance to AJ [64]. The processes that are related to plant growth involve the AUX in the biosynthesis of IAA [72]. PGPRs that occupy plant roots contribute to plant growth and the synthesis of CKs, ABA, gibberellic acid, and IAA [73]. CKs are related to AUXs both synergistically and antagonistically in maintaining the physiology and growth of the vegetable [74].

The BRs are hormones that also contain bioactive molecules (brassinolide) with the function of natural endogenous ligands in dicotyledons. Such correlation promotes a phosphotransfer cascade, providing a nuclear increase in transcriptional effectors in the resulting direction, modulating the expressions of the genes of interest [75]. The communication between plant roots and nearby soil occurs through chemical signaling that preserves the viable conditions of plant cells and their physical properties, providing better conditioning for their growth. Plants use root exudates to improve nutrient absorption, similar to rhizodeposition (dead vegetable tissues, leaves, and root cells) which also influences soil microorganisms [76].

### 6.2. Nitrogen Fixation

Rhizobacteria are the main contributors to atmospheric nitrogen fixation in plants. In this way, these bacteria encourage the expression of nod genes by recognizing signals from plants, unique to each rhizobium, which biosynthesizes lipo-chito-oligosaccharides (chitin), which are structural components of Nod factors. Plants act by identifying rhizobia as phytopathogens and thus incite defense genes, phytoalexins, and ROS through different mechanisms (lipopolysaccharides—LPS) and effector proteins secreted by type-III secretion system (TTSS). Consequently, the plants distinguish symbiotic partners and consent to nodulation. The synthesized Nod factor binds to the Nod receptor present in legumes in the plasma membrane of root cells, which undergoes a structural transformation where the rhizobium enters the plant cell by endocytosis [77].

Similarly, endophytic fungi (AMF) can assist in the nitrogen cycle, providing an increase in the ability of rhizobia and azospirillum bacteria to fix nitrogen. As the stages of the nitrogen cycle consist of denitrification, N fixation, nitrification, ammonium oxidation, and finally ammonification, AMF acts in denitrification and co-denitrification [71].

### 6.3. Phosphate

Phosphorus typically is detected in the soil in its organic (P_o_) and inorganic (P_i_) forms. This compound can be replaced through the mobilization of P_i_ or biological procedures of P_o_ mineralization, with the genes being related as phoD (alkaline phosphatase), phoC (acid phosphatase), and phnX (phosphonatases). The specific phosphate transporter (pstS) genes are responsible for increasing microbial mastery of phosphorus perception when there is a shortage of the compound [78].

At low concentrations of phosphorus, plants usually form thin, long, and creeping roots, using physiological characteristics to absorb higher amounts of the compound. Such particularities involve carboxylates, and acid phosphatases in addition to the release of flavonoids through symbiosis with MFs. When releasing phosphatases and flavonoids, the enzyme hydrolyzes P_o_, releasing P_i_ for plants and microorganisms that absorb only the inorganic compound. Flavonoids mobilize phosphorus by releasing compounds added to Fe and Al [79].

Arbuscular mycorrhizal fungi (AMF) have a preference for phosphorus from soils over roots, where through soil exploration, they generate organic acids and phosphatases aiming to activate low-solubility phosphorus, absorbing the available compound with high affinity for the phosphorus transporter [80,81]. AMFs share the rhizosphere with phosphorus mobilizing bacteria (PMB), producing a triad of holobionts between “plant—AMF—PMB”, with emphasis on the genes involved with PMBs (phoD and phoC), which act in the mineralization of P_o_ (or gcd) and pqqC for P_i_ solubilization [81].

### 6.4. Siderophores

Iron is a component of substantial importance in plant development. When there is a shortage/deficiency of this compound, microorganisms (fungi and bacteria) and plants are capable of producing a secondary organic metabolite of low molecular weight (<10 kDa) known as siderophores. They are chelating agents secreted in the extracellular environment, showing high affinity with the metal. Examples of siderophores include catecholates (or phenolates), hydroxamates, carboxylates, and mixed compounds (which have more than one functional group) [82,83].

The siderophores produced by plants are known as phytosiderophores. There is a relationship between the microbial siderophore and the phytosiderophores, expressed regarding their functioning process, where the plant metabolites trap the insoluble Fe^3+^ present in nature as Fe(OH)_3_ or Fe_2_O_3_. In the sequence, the compound recognizes the group formed by proteins or siderophore receptors that are on the surfaces of external membranes [84]. After its identification, the complex is sent to the cytosol, where the Fe^3+^ is converted into soluble Fe^2+^, thus being accessible to the microorganisms [82].

Siderophores also exhibit a mutable structure, indicating their non-selectivity for a single metal ion. As a result, they can be complex with other metals and metalloids, such as copper (Cu), nickel (Ni), and zinc (Zn) present in the environment. In another context, this specificity allows siderophores to facilitate the separation of metals and metalloids from nutrient solutions while also contributing to microbial resistance. Once chelated, metals and metalloids can be extracted through biosorption and bioaccumulation [85].

The production of siderophores by bacteria is exclusively extracellular. Both gram-positive and gram-negative bacteria and aerobic and facultative anaerobic bacteria produce the metabolite in circumstances of stress due to lack of Fe. Another difference involves the synthesis of extracellular and intracellular siderophores by fungi [82]. Endophytic fungi can produce different types of siderophores, such as ferrichromes, coprogens, and fusarins. Another specificity of endophytic fungi is in the structure of the siderophore, where ornithine groups are commonly found, unlike endophytic bacteria which are characterized by acylated alkylamine structures [71]. The fungus *Trichoderma harzianum* isolated from the inflorescence of *Aloe vera* L. showed good results in both growth and siderophore production in plant growth assessments. The fungal endophytes can prevent the development of phytopathogens by restricting Fe availability, negatively interfering in the generation of nucleic acids, and sporulation of phytopathogens, cooperating with the biocontrol function of fungal endophytes [69].

### 6.5. Volatile Compounds

Quorum sensing (QS) molecules used in signaling between microbial communities were discussed to synchronize and promote better inter- and intraspecific responses. Molecules produced by bacteria and fungi were described, which require detailed studies to understand the best way to act as signalers. There are volatile organic compounds (VOCs) produced through different pathways of metabolite processes, presenting low molecular weight, in addition to their lipophilic characteristics, significant for long-distance communication between microbial communities. This signal is cognate both among the microorganisms present in the community and communication with the plant in the rhizosphere. Examples of molecules produced by bacteria and fungi include terpenoids, alkanes, alkenes, ketones, alcohols, and sulfur-containing compounds. These compounds can act in signaling like QS and in the chemical inhibition of antimicrobial activities. They influence gene expressions that are related to hormonal signaling, defense pathways, and compliance with biotic stresses, virulence, and biofilm generation [51].

In a study involving *Trichoderma* spp., da Silva et al. [86] verified the production of VOCs that were effective in suppressing phytopathogens, causing plant growth. VOCs involve acetic acid, dimethyl disulfide, 6-pentyl-α-pyrone, dibenzofuran, and methanethiol. The compounds belonged to the polyketide class, known to be agents that inhibit the mycelial development of phytopathogenic fungi. A summary of these plant growth promoters is presented in Table 1.

## 7. Plant Roots

Considering that the rhizosphere consists of a region of the soil that surrounds the root, it is expected that this location will be impacted by it. Root intervention occurs both physically through the generation of heat and chemically through the secretion of metabolites from plants by an active energetic pathway instigated by ATP hydrolysis or diffusion [116]. In this process, plants provide the soil substances of low (amino acids, sugars, phenols, terpenoids, and lipids) and high molecular weight (proteins, polysaccharides, and nucleic acids) that are consumed by the microbiota according to the growth stage involved, where such distribution occurs depending on the chemical properties present [117].

The interactivity between plants and microorganisms in rhizospheric environments usually occurs by microbial development within or around the roots, enabling cooperation that can be identified as symbiotic, neutral, or parasitic. This condition also occurs through the nutrition of the plant by the soil, the surrounding soil, the plant’s protection method, and the microbial genus/species that live in the rhizospheric extension [118].

Similar to the aerial parts, vegetable roots are important factors for the plant’s immunity as they have a higher microbial solidity. The rhizosphere presents considerable quantities of microorganisms with a typical representation of 10^6^ to 10^9^ spores (mL bacteria)^−1^, 10^5^ to 10^6^ spores (mL fungi)^−1^, and 10^1^ to 10^2^ spores (mL of nematodes × gram of soil)^−1^ [116]. Some of the characteristics of this microbiota focus on the growth impulse of plants, such as rhizobacteria, but also because they present pathogenic characteristics such as the fungus *Fusarium* spp., reinforcing the relevance of roots as an entry route for opportunistic organisms through epidermal fissures between their main and lateral channels, as well as in young roots that are devoid of secondary cell walls [2].

The mechanism of action of rhizospheric microorganisms in the soil surrounding the roots of plants occurs through root exudates, which include sugars, amino acids, organic acids, phenolic compounds, enzymes, phytohormones, and vitamins. These exudates alter soil chemistry, creating a selective environment where microorganisms with greater adaptability prevail. Root exudates can either attract or repel microorganisms, influencing their association with the plant. These interactions can be beneficial, characterized by mutualism or symbiosis, or detrimental, involving competition, parasitism, or pathogenesis. The relationship considered favorable is separated regarding nutrition, which encourages plant growth indirectly, preventing the action of phytopathogens, and through direct stimulation by phytohormones [119]. Plants are subordinated to the dexterity of transmitting information between rhizospheric microorganisms. Endophytic, symbiotic, and pathogenic microorganisms are soil microbiomes that have obtained a higher amplitude [118].

Another manifestation of plants in the presence of pathogens consists of ROS hyperproduction, a toxic component that stimulates certain signaling, causing modifications in the plants’ gene expression patterns, and inciting vegetable resistance or susceptibility to pathogens [120]. Vascular pathogens enter through the roots and can cause symptoms in the airways of plants, such as anthracnose in corn from *Colletotrichum graminicola*. Despite the infection being through the roots, it is not considered a root disease pathogen.

Even though the root system is complex, and most soil microorganisms are not capable of being cultivated in laboratories (which commonly results in studies focused on plant aerial parts), is essential to detect pathogens present in the soil so that is possible to initiate defense responses and prevent the spread of the disease [2]. Plant-beneficial rhizospheric microorganisms (PBRMs) are important components for plant growth due to their good performance in terms of solubilization and nutrition, stimulating kinesis and nutritional clearance of plants through chelating agents, acidification, and redox modifications [117].

The main relationship between microorganisms and plant roots occurs through association with bacteria or rhizobacteria (plant growth-promoting rhizobacteria, PGPR). This affinity is classified according to the location in which the microorganism is found, such as symbiotic bacteria present inside plant cells and promoting nodules as atmospheric nitrogen fixers. In alfalfa and lima beans, *Sinorhizobium meliloti* and *Rhizobium leguminosarum* are usually found, respectively. Another place where such bacteria are found is free-living, in the plant cell, being responsible for plant growth, although they are not nodule promoters, such as *Azobacter*, *Azospirillum*, *Bacillus*, and *Klebsiela* spp. PGPRs also provide the solubilization of inorganic phosphates, producing phytohormones such as AUX, CKs, and GAs, which favor Fe nutrition through chelators (siderophores), the synthesis of antibiotics, in addition to competition for space and nutrients in plant defense [119].

IAA is produced through its precursor tryptophan present in root tips, which control and metabolize ET through the enzyme aminocyclopropane-1-carboxylic acid (ACC) deaminase [51,121]. It breaks down the ethylene precursor into ammonia and α-ketobutyrate, inhibiting plant growth [122]. PGPR is also responsible for inciting ISR, with *Pseudomonas* being the best-known strain, which does not cause visible damage to the plant’s root system. In this induced resistance, the main phytohormones involved are AJ and ET. Such induction can also occur through interaction with aerobic, non-pathogenic bacterial microorganisms, or even artificially stimulated by chemical agents. ISR is involved with β-1,3-glucanase, phenylalanine ammonia-lyase, peroxidase, chitinase, chitosan, and polyphenol oxidase [58].

Another strain belonging to PGPRs is *Bacillus*, where Gamez et al. [123] report the efficiency of the fungus *B. amyloliquefaciens* Bs006 in colonizing banana roots (*Musa acuminata Colla* cultivar Williams) as a biofertilizer, providing good results for the plant’s growth and development. Li et al. [124] evaluated the use of *B. thuringiensis* as biological control agents (BCAs) in plant parasitic nematodes through its active component, an ingestible crystal protein (Cry5B) that is toxic to *Caenorhabditis elegans* present in tomato roots (*Lycopersicon esculentum*). *Bacillus amyloliquefaciens* CM-2 and T-5 were applied to tomato seedlings, offering satisfactory results for the biocontrol of the pathogen *Ralstonia solanacearum*, which causes vascular wilt [125].

Unlike bacteria, the plant’s relationship with fungi occurs through mycorrhizal fungi (MFs) through the symbiosis between root and endophytic organisms. This correlation occurs through mycelial fungi, capable of colonizing up to 80% of angiosperms and all gymnosperms, also providing phosphorus, water, micronutrients, root expansion, and secondary metabolites to the plant, while the fungus is reciprocated with carbon sources [119].

The FMs usually have a mutualistic relationship with plants and are generally used as biostimulants since they increase the absorption of nutrients such as P and N, whereas in some cultures their tissues typically have a higher load of certain micronutrients such as Cu, Zn, Fe, and Mn [51]. However, for the interaction between fungus and plant to be mycorrhizal, some harmonious requirements regarding the development of the host are necessary, whether in its classification, structure, or functional requirements.

Ectomycorrhizae (ECTO), Endomycorrhizae (ENDO), arbuscular mycorrhizae (AM), ericoid mycorrhizae (EM), or orchid mycorrhizae (OM) are some classifications. In terms of its structure, the formation of a Hartig network and mantle (ECTO) or arbuscules, spirals, and platoons (ENDO) can occur; in addition to functional requirements, which allows for unimpeded nutrient supply and management of host development [126].

ECTOs correspond to symbiotic associations between fungi (phylum *Basidiomycota*, *Ascomycota*, and *Mucoromycota*) and species of land plants in a percentage of up to 2%, where the main examples are pine and oak [119]. This interaction occurs through an extracellular mycelial mantle that comprises the Hartig network around the tips of the plant’s roots, where the sharing of nutrients and C takes place (in the case of ENDO, its functionalities are associated with the internal part of the roots) [127].

Both AMs and EMs conceive inter- and intracellular structures with the roots. This process is designed in a different format, where the AM presents an arbuscule shape and the intracellular structures of the EM intertwine in the branches of the fine roots. Hawkins et al. [127] described that plants from AMs correspond to 70% of herbaceous and woody species where the characteristic phyla of fungi are *Glomeromycota*. Regarding EMs, there is the involvement of two phyla—the *Ascomycota* and the *Basidiomycota*—where their interaction refers to 1% of plant species, such as the ericaceous plants of the *Ericaceae* family. OMs involve the phylum *Basidiomycota*, corresponding to most orchid species. This association is linked to the plant’s dependence on the endophytic fungus in its initial phase of development to obtain nutrients and carbon substrate, a process known as myco-heterotophic, where in this situation the fungus acquires C through adult photosynthetic plants.

The interaction of endophytic fungi with roots occurs, having in this process the creation of a diversity of microorganisms known as endophytic filamentous fungi (EFF). These fungi are identified in most plants and their variations range from the plant genotype to the accessibility to nutrients depending on the species of microorganisms present in the environmental conditions. Part of these EFFs are classified as clavicipitaceous endophytes (related to grasses) and non-clavicipitaceous endophytes (related to non-vascular plants, conifers, and angiosperms). As a highlight, there is the phylum Ascomycota, followed by *Basicomycota*, *Zygomycota* (also known as *Mucoromycota*, since *Zygomycota* is usually considered a polyphyletic group), and *Glomeromycota* [126], where its colonization can occur both vertically (by seeds) and horizontally (colonizing the host).

Endophytic fungi of the phylum *Mucoromycota* interact with the roots and rhizoids of vascular or non-vascular plants, where such interaction acts as a functional mutualism. Unlike dark septate endophytic organisms, the phylum *Mucoromycota* is related to a certain taxonomic category between fungi and roots, of which the hyphae act inter- and intracellularly, being then melanized, although there is no certainty of functional mutualistic interaction [127].

Fungi are usually divided into three classes of interaction with plant growth: positive, neutral, or negative interaction. The so-named neutral class does not have relevant impacts on the plant’s development, unlike positive and negative interactions, where the latter has antagonistic applications, giving fungi a pathogenic classification with the species of genera *Fusarium*, *Pythium*, *Aspergillus*, *Botrytis*, *Puccinia*, and *Rhizoctonia* through the production of toxins. Plant-growth-promoting fungi (PGPF), such as *Trichoderma* spp., have been used to eliminate such negative follow-up, considering their high reproductive demand, their ability to produce secondary metabolites, and the inhibiting of the development of pathogenic fungi. These microorganisms are ubiquitous, non-virulent, and opportunistic plant symbionts, possessing the ability to live under parasitism in other fungi. They colonize plant roots by entering their epidermis, a process that promotes an increase in the synthesis of enzymes such as β-peroxidase, chitinases, 1,3-glucanases, and hydroperoxidase lyase from the lipoxygenase pathway [10].

A good performance of the *Trichoderma* species regarding the control of white mold caused by *Sclerotinia sclerotiorum* was reported, inhibiting 81.5% of its in vitro action on cucumber plants. The use of the species in question as a PGPF in biological control is highlighted due to its performance both as antibiosis and as myco-parasitism, a process that induced its resistance [128].

## 8. Interaction Between Phytopathogenic Microorganisms and Plants

Due to the varieties of plant species, each one can be affected by a type of pathogen (fungus, virus, bacteria, or oomycetes) with different forms of infection and proliferation and, consequently, different host reactions to microbial penetration. Likewise, the same phytopathogen can attack different plant species [57].

### 8.1. Fungi and Oomycetes

The microbial physiology of the fungus establishes a distinction regarding its cell wall, which differentiates it from saprotrophic microorganisms and opportunistic pathogens in host infections. This differentiation allows the cell wall of the microorganism to play a crucial role in its infection, where the release of chitin and β-glucan oligomers indicates that the infection was not successful due to the action of the host’s chitinases and glucanases. The fungus’ perception of host manipulation occurs through indications provided by the host, which may be through the development of exclusive infection systems, such as appressoria, or by the configuration of feeding through haustoria. The plant’s defenses suffer from lower levels, evading identification by its immune system [129].

Fungi and oomycetes, phytopathogenic or symbiotic, typically proliferate in plants through the haustoria of the host’s plasma membrane, extending hyphae to the top between or through plant cells (an intercellular mechanism) [20]. These microorganisms can also penetrate the plant through the appressoria, thus being specific leaf infections [130]. The interaction between fungi, oomycetes, and plants occurs through apoplasts composed of secondary metabolites and hydrolytic enzymes that prevent both the growth of the fungus and the oomycete, a microbial process favored when the apoplast is neutralized by effectors [131]. Thus, the extracellular or apoplastic effectors of oomycetes interfere in plant defense by inhibiting the action of proteases and glucanases, translocating plant cells, and affecting the virulence of the plant [132]. However, some intracellular effectors move into cells, such as RxLR (located within the cytosol) and Crinklers (cytoplasmic effectors) [133].

Fahrentrapp [57] reports data concerning the fungus *Botrytis cinerea*, which has necrotrophic representation and simultaneously presents life as an endophytic organism in lettuce, initially developing in the root and later extending to the rest of the plant. *Beauveria bassiana* is described as an endophytic microorganism in some species of corn (*Zea mays*), potato (*Solanum tuberosum*), cocoa (*Theobroma cacao*), coffee (*Coffea arabica*), cotton (*Gossypium hirsuutum*), and soybean (*Glycine max*) [133]. This process can also be used against phytopathogens, such as in the intervention of basal rot affected in onions caused by *Fusarium oxysporum* f. sp. *cepae*, as well as in the treatment of *Rhizoctonia solani*, which causes seedlings to fall over and root rot in older plants [134].

*Ganoderma boninense*, which causes white rot, also causes stem rot in oil palms (*Elaeis guineesis jacq*., from the *Arecaceae* family). Chow et al. [134] evaluated the use of BCAs as an alternative using *Trichoderma* spp. (*Trichoderma harzianum* and *Trichoderma viride*), *Gliocladium viride*, *Diaporhe phaseolorum*, *Pseudomonas fluorescens*, *Penicillium* spp., *Burkholderia* spp., and *Bacillus* spp. The endophytic microorganisms *Diaporhe phaseolorum*, *Trichoderma asperellum*, and *Penicillium citrinum* presented efficiency in triggering the defense of palm trees after the pathogenic infection.

### 8.2. Bacteria

The propagation of bacteria in plants can occur through intercellular spaces (apoplasts) after entry through gas pores (stomata) or water (hydatodes), or by superficial lesions. Similarly to fungi and oomycetes, the bacteria can exclude plant defense responses and introduce effectors [135]. To reduce the harmful effects of pathogens, biocontrol agents such as plant growth-promoting bacteria (PGPB) promote healthy relationships with plants, encouraging their development through indirect procedures, such as nitrogen fixation and mobilization of nutrients (Fe and P) through the production of siderophages and organic acids. Aiming to curb phytopathogens, PGPB promotes disputes for space and nutrients, such as antibiosis, where a typical example is *Pseudomonas*, which produces antibiotics such as pyoluteorin and 2,4-diacetylfloroglucinol in the competition against tobacco root rot caused by *Thielaviopsis basicola*. Pyoluteorin and pyrrolnitrin are used for watercress disease caused by *Pythium ultimum* and *Rhizoctonia solani*. Also, 2,4-diacetylphloroglucinol is used for wheat disease caused by *Gaeumannomyces graminis* [136].

There is the stimulus of the lytic enzymes production, such as chitinases for black rot caused by *Xanthomonas campestres* (proteases and cellulases) against *Rhizoctonia solani*, *Pythium aphanidermatum*, and β-1,3-glucanases. Inhibition of toxins and the stimulation of plant defense techniques, such as ISR, exist [135]. Bacteria that favor plant growth are endophytic microorganisms, also known as endophytic plant growth-promoting bacteria (EPGPB). They are used against pathogens in pre- or post-harvest periods, being the first induction of prophylaxis in phytopathogens of agricultural soils [136].

## 9. Bioinputs

Agricultural pesticides consist of substances, initially natural and from organic materials, which have been replaced by inorganic and synthetic compounds aimed at containing living organisms that cause damage to crops [137]. The compounds include herbicides, insecticides, fungicides, bactericides, nematicides, molluscicides, and acaricides, being widely used in agricultural systems along with fertilizers to increase soil nutrition [138].

Mandal et al. [138] described that the high demand for chemical compounds is associated with the increase in the consumption of nutrients from the land in addition to climate alterations and urbanization that are inevitably limiting the availability of arable lands for agriculture. Panda and Zhou [139] described that the undesirable aspects resulting from the use of agrochemicals on the environment and ecosystems have become noticeable, causing the destruction of beneficial microorganisms in the soil and, the resistance of plagues to pesticides, and leaching of chemicals into bodies underground or surface hydric sources. Imminent environmental pollution, a threat to the health of farmers, and danger to the natural cycle of plants and animals due to the unrestrained spraying of toxic components are also seen [140].

One of the scientifically suggested alternatives for biological control of agricultural management is the use of bioinputs. They originated from biological sources, animal microbial production (product, process, or technology), and plant material. These compounds have the function of enhancing agricultural and aquatic production, ensuring their quality [141]. Biological inputs can be used in nutrient management and control of diseases caused by pathogens and phytopathogens, contributing to profitable, eco-efficient, and health preservation values [142]. Two concepts are used: biostimulants, which are substances or microorganisms that stimulate/facilitate crop nutrient absorption, optimize the plant’s physiological processes, and increase stress tolerance; and bioinoculants, which are biological products containing beneficial microorganisms that directly promote plant growth and induce resistance. They can be applied to the soil or plants and include categories such as biofertilizers [143].

### 9.1. Biostimulants

Even in small quantities, the biostimulants can improve the plant’s physiological system, being responsible for stimulating it to favor the quality and efficiency of crops in the absorption of essential elements [144] The components enlarge the plant’s tolerance to biotic and abiotic stress, increasing the availability of nutrients and their solubilization through alterations in the vegetable’s root structure or by improvements in the enzymatic and microbial activities of the soil [145].

Biostimulants do not promote toxicity to non-target organisms, presenting low ecological constancy. They do not promote negative effects throughout the cultivation process. In comparative terms, biostimulants differ from chemical fertilizers due to their incitement of adaptive cellular responses without necessarily making use of mineral elements in their compositions. They can be applied as a complement to fertilizers and phytopharmaceutical products [139].

**Figure 3:**
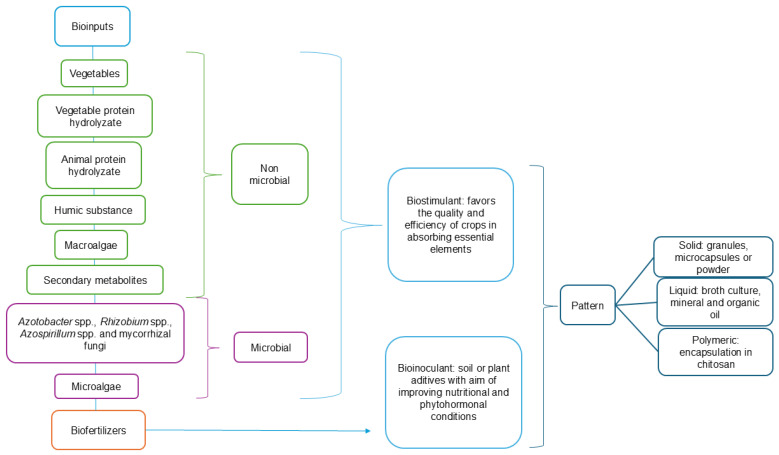
Chemical differences between bioinputs in plants and their structure/formulation before the dispersion phase.

Enzymes, phenolic compounds, amino acids, hormones, saccharides, peptides, proteins, organic components, hydrolyzed-derivatives of proteins (signaling peptides and free amino acids), humic compounds, and microorganisms (bacteria, yeast, filamentous fungi, and microalgae) stand out [146]. However, the European Commission through Regulation number 1009 of 2019 establishes that biostimulants of microbial origin are named rhizobacteria and arbuscular mycorrhizal fungi, while the non-microbial components are algae extracts, hydrolysates of vegetable and animal proteins, humic substances (fulvic and humic acids as well as humins), chitosan, and inorganic compounds (phosphites and silicon) [147]. Figure 3 presents the chemical differences between the bioinputs in plants and their formulation before the dispersion phase.

#### 9.1.1. Non-Microbial Biostimulants

Vegetable biostimulants can be obtained from industrial byproducts where knowing the biochemical processes involved between plants and these compounds can contribute to the development of agronomic methods. Biostimulants control the biosynthesis of secondary metabolites that permeate the fortification and defense of plants [145]. However, biostimulation by plants presents variations and interferences; that is, its effectiveness tends to vary according to the crop involved, such as fruits in annual and ornamental species and vegetables produced in greenhouses. The variations extend to the occurrence of stimuli from previous times, such as the displacement of organic or inorganic tree reserves, and the inconsistency of factors associated with climate changes [148].

Plant biostimulants improve the nutritional capacity of the vegetables by increasing the absorptive surface of the roots. Extracts of *Moriga oleifera* (Lam.), help various plant crops, favoring their growth caused by the increase in the content of minerals, amino acids (proline), phytohormones (cytokine, auxin, and gibberellin), and antioxidants [148]. Del Buono [143] showed favored growth of pumpkin (*Cucurbita pepo* L.) towards water stress. Tomatoes (*Solanum lycopersicum*) showed benefits to plant functioning when *Moringa* leaf extracts were applied exogenously during their cultivation, highlighted by the high content of cytokinin and benzylaminopurine [149].

There are also vegetable protein hydrolysates such as legume seeds, lupins, and caseins that contain bioactive substances (amino acids—aspartic acid and glutamic acid—essential amino acids, and peptides—oligopeptides and polypeptides) [150]. Another example is residual biomasses from crops and agricultural by-products, which stimulate plant microbiota during vegetable development, improving carbon and nitrogen metabolism and nutrient accessibility [144,151].

Residual biomass can be applied as foliar or as a growth substrate, allowing different harmonious results at molecular, physiological, and biochemical levels according to the chosen application method [144]. Thus, one of the advantageous results of the application of vegetable hydrolysates is linked to the bioactive peptides that are similar to the hormones auxin and gibberellins, apt to promote alterations in plant roots, and in aerial parts, providing improvements in nutrient absorption and yield of the culture [151,152].

Animal protein hydrolysates such as poultry waste feathers, containing large amounts of proteins, can also be used as biostimulants [153]. Hydrolysates from animals have compounds in their structure that are auxin precursors, such as tryptophan and phenylalanine, and signaling peptides responsible for inciting the plant’s resistance system against salinity, water, and thermal stress. There are heavy metals and other compounds that stimulate the synthesis of chlorophyll, increasing its photosynthetic efficiency [154]. Del Buono [143] described the use of biostimulants from animal hydrolysis in tomatoes during periods of drought, reporting that the animal hydrolate induced an increase in the levels of indole-3-acetic acid, trans-zetaine, and jasmonic acid.

The soil organic matter has a fundamental and active component: the humic substance. Humus is composed of heterogeneous biomolecules with recalcitrant and stable characteristics generated in natural soil, sediments, and aquatic ecosystems [150,155] recognized for inducing modifications in the physiology and structure of plant roots, providing better absorption of nutrients. Humus is divided into three parts according to its solubility and different pH conditions: humic acid (HA), soluble at pH above 2; fulvic acid (FA), soluble at any pH and; humins (HU), soluble in water [150,154,155].

HA and FA are more active substances than soil HS, being significantly related to soil improvements, mainly in fertilization control, buffer conditioning, and maintenance of acidity and alkalinity [155]. HA stimulates plant development in the presence of abiotic stresses, promoting beneficial alterations in both primary and secondary metabolism, as well as in antioxidant activities. Del Buono [143] described data about the combination of FA with superabsorbent polymers (the first applied to the plant crowns and the second to the soil) in corn crops during periods of drought, obtaining a decrease in leaf transpiration through the stomatal openings, promoting grain productivity and the abscisic acid content of the leaves.

Marine algae or macroalgae also have biostimulant potential, being heterogeneous products where their extract diversifies depending on the species used (brown, green, or red seaweed for example), the techniques used to acquire the vegetable biostimulant [144]. Among the substances present in seaweed extracts include amino acids, polysaccharides, vitamins, fatty acids, minerals, phenolic compounds, and traces of phytohormones [146].

Biostimulants based on the brown macroalgae *Ascophyllum nodosum* resulted in improvements and increases in polyphenol levels and the gene induction of water scarcity in the treatment of *Arabidopsis thaliana* [155]. Red macroalgae *Kappaphycus alvarezzi* applied to corn roots in periods of drought promoted a higher volume of lateral root biomass, favoring nutrient absorption [144]. This factor is associated with the beneficial characteristics of micro- and macronutrients of algae, and the presence of phytohormones (auxin and cytokine), salts, and compounds such as glycine betaine, and choline.

Secondary metabolites are also plant root growth stimulators. These compounds are differentiated according to their biosynthesis (alkaloids, phenylpropanoids, steroids, terpenoids, polyketides, lipopeptides, amino acids, and fatty acids), having low molecular weight. They can act as signaling devices (such as phytohormones and siderophores), promoting symbiosis with beneficial microorganisms to plants and helping the plant against biotic and abiotic stresses. Extracts of sorrel (*Rumex acetosella*) are mentioned, which are beneficial against the harm caused by barley powdery mildew, and phenolic compounds extracted from the macroalgae *Ecklonia maxima* (such as phloroglucinol (benzene-1,3,5 triol, PG) and eckol (4-(3,5-dihydroxyphenoxy) dibenze-p-dioxin-1,3,6,8-tetrol)). They are stimulators of the development of *Eucomis Autumnalis* (pineapple flower) and the rooting of corn seedlings (*Zea mays*). Treatments with pyoverdine (a siderophore generated by *Psedomonas fluorescensi* in tomato seedlings (*Solanum lucopersicum*) through the addition of ferric ions were able to increase plant development (within the necessary iron limits) the synthesis of photosynthetic pigments, carotenoids, and anthocyanins [149].

#### 9.1.2. Microbial Biostimulants

Microbial biostimulants are non-pathogenic and toxicogenic, covering the fungi *Azotobacter* spp., *Rhizobium* spp., *Azospirillum* spp., and mycorrhizal fungi [156]. These microorganisms can increase the nutritional condition of the plant, providing a higher volume of soil reachable by its roots (through mycorrhizae), enabling the absorption of previously unavailable nutrients, such as N-fixing microorganisms and those capable of solubilizing P, Fe, Mn, and Zn [148]. Arbuscular mycorrhizal fungi have better functionalities in nutrient absorption, transfer, and incorporation of macro- and micronutrients, and increased photosynthesis and control of both plant hormones and the variation of microbial communities in the rhizosphere [144].

Examples of microorganisms that can act in the formulation of bioinputs are the *Trichoderma*. They are available in soils correlated to the plant rhizosphere and distributed in an endophytic relationship with the plants, demonstrating benefits in biocontrol in crops through their growth, antagonizing, and competing with phytopathogens present in the rhizosphere [157]. These microorganisms can produce volatile compounds such as 6-pentyl-2H-pyran-2-one and auxins, which help in the development and branching of plant roots [139], and the solubilization of phosphates, the production of siderophores, enzymes, phytohormones (gibberellins, cytokinins, and ethylene), and secondary metabolites [157]. *Bacillus* ssp. and *Pseudomonas* ssp. also favor the modulation of plant development through volatile organic molecules, such as terpenes, N-acyl-l-homoserine lactonones, and cyclopeptides [139].

Microalgae are microscopic organisms from various photosynthetic groups, especially the unicellular ones that operate under sunlight and CO_2_ to synthesize metabolites. Blue-green algae (or cyanobacteria) have the characteristics of N fixation in rice fields and for other crops. They are potential biocontrol organisms as they incite enzymatic activity in the plant defense system and produce antimicrobial substances when attacked by phytopathogens. Microalgae are little applied in agricultural production, compared to seaweed, and are mainly manipulated in the animal feed, bioremediation, and cosmeceutical products sectors. A way to use microalgae in bioremediation is to apply its biomass directly to the soil. Although it is a leisurely process, it allows an improvement in the physical, physicochemical, and biological conditions of the land [146].

### 9.2. Bioinoculants

Bioinoculants are microorganisms from biotic and abiotic resources, that is, from microbial isolates such as fungi, bacteria, certain nutrients, and organic or inorganic transporters. They are classified as soil or plant additives to improve their nutritional and phytohormonal conditions and control pathogenic organisms, alleviating both biotic and abiotic stresses [158]. Bioinoculants are also capable of producing biofilms, ACC deaminases, siderophores, EPS, antibiotics, antioxidants, and hydrolytic enzymes (protease, cellulase, chitinase, and β-glucanase). They also act in the solubilization of minerals (P, K, and Zn), fixation of N, cryoprotectants, volatile compounds, remediation of heavy metals by bioaccumulation, biotransformation, or biosorption and induction of resistance, whether induced (ISR) or the acquired (SAR) resistance [122].

The microbial bioinoculation in the soil can interfere with the present microbiota, decreasing, increasing, or even having no effects on soil taxa. Microorganisms need to be in a uniform matrix or on a carrier, as represented in Figure 3**,** providing them with good conditions for their development. It allows them to act as desired, that is, making it possible to reserve, move, and preserve without polluting the environment.

The carriers can have different formulations, solid, liquid, and polymeric. They have non-toxic characteristics and are sterilizable, capable of providing necessary and adaptable nutrients to different microorganisms. Solid formulations can be in the form of granules, microcapsules, emulsions, or powders [126]. Liquid formulations are based on cultures in broth, minerals, organic oil, emulsions, or suspensions based on a polymer [159]. The application of these components may vary according to their formulations, whether by irrigation (or spraying) systems, seed coating, or centrifuge spreading. There is still the method of soaking transplanted crops, such as rice, onions, and ornamental plants. Biostimulants also come in natural solid formulations, such as peat and coal, soybean oil, or inert solids, such as perlite, talc, and clay [125].

Sharma et al. [71] described that the interaction of the plant with its endophytic microbial community (endobiome) can occur intracellularly or intercellularly. Some endophytic fungi are from groups classified as polyphetic, with emphasis on the phylum Ascomycota and the genus *Fusarium*, *Aspergillus*, *Alternaria*, *Trichoderma*, *Penicillium*, *Cladopsorium*, *Colletotrichum*, and *Talaromyces*, which are capable of producing bioactive secondary metabolites (alkaloids, quinones, steroids, saponins, tannins, and terpenoids). They can be used as bioinoculants acting as biofertilizers.

FMs can be used individually or in co-inoculation with other microorganisms. Co-inoculation occurs between FM and bacteria due to the existence of regulation for this association, and the performance of the BCA biostimulant for the co-inoculated bioinoculant. The bacteria most used in this process are those with N-fixing, phosphate-solubilizing, and rhizobacteria characteristics, highlighting *Bacillus licheniformis*, *Bacillus subtilis*, *Bradyrhizobium japonicum*, *Pseudomonas fluorescens*, and *Rhizobium meliloti*. Such microorganisms also can produce antibiotics and fungal cell wall degrading enzymes, and induce resistance when used in the BCA [126].

Endophytic bacteria have potential as bioinoculants, with emphasis on *Pseudomonas*, *Bacillus*, *Burkholderia*, *Stentrophomonas*, *Pantoea*, and *Mycobacterium*, which promote plant growth [160]. They produce metabolites capable of directly intervening in the physiology and biochemical processes of the plant, providing the mobilization of nutrients and the production of biomolecules and hormones. Indirectly they influence when the process involves the action of microorganisms as biocontrol agents, safeguarding the plant via microparasitism, lack of nutrients, hydrolytic enzymes, toxins, and induced resistance [71]. Therefore, the selection of a suitable bioinoculant must involve a thorough analysis of affordable costs, easy handling, and application, and efficiency throughout its useful life [126]. The use of bioinoculants is a recent technique in the agricultural sector, still requiring constant innovations aimed at its improvement.

#### Biofertilizers

The preparation of biofertilizers consists of some stages such as (i) microbial screening (strains isolated from soil, rhizosphere, and plant tissues); (ii) investigation of the functional characteristics of the biofertilizer (selected crops are studied according to their functionality—nitrogen fixation, nutrient mobilization, phytohormone production, among others—and tested in greenhouses); (iii) product formulation (selection of the carrier to be used—liquid or solid); (iv) cultivation of microorganisms selected from the screening step (characteristics and fermentative methods); and (v) testing the formulation and evaluating its efficiency (large-scale field test) [161].

Knowing the elaboration process, biofertilizers are composed of microorganisms. Kapoore et al. [145] described that biofertilizers are ecological resources constituted of microorganisms (fungi, microalgae, and bacteria) capable of colonizing the rhizosphere, allowing higher absorption of N, P, and K (nitrogen fixation, phosphorus solubilization, and potassium mobility), solubilization of Zn, and oxidation of sulfides and minerals that help in the development of the plant. Unlike organic fertilizers (animal manure and slurry residues), biofertilizers may contain one or more combining microorganisms apt to settle in the rhizosphere and promote direct or indirect benefits for plant development [159]. The difference regarding biostimulants occurs in the ease with which the latter provides the nutrients’ absorption, related to their physiology, in the plant’s functioning process [146].

In addition to improving the physicochemical properties of the soil, biofertilizers are capable of promoting alterations in the microbial structure and function, such as modifying the C load, and the diversity and physiology of the microbial community. Not all microorganisms interact with biofertilizers in the same proportion, sometimes requiring the addition of PGPRs, affecting the present microbial community [159]. They are bioindicators of ecosystem functioning, given that soil microbial communities have a high degree of biodiversity. In this scenario, biofertilizers contribute to the sequestration of CO_2_ from the atmosphere, increasing the C content in arable land and restoring soil health. This process fits into regenerative agriculture or can even be denominated “sustainable green agriculture” when biofertilizers come from microalgae [161].

Another way to characterize biofertilizers is their formulation, which can be solid, encapsulated in polymers, liquid, or even in dry form in fluidized beds. Mącik et al. [161] described the immobilization of the fungus *Azospirillum brasiliense* Ab-V5 in biodegradable foams and *Pseudomonas putida* A (ATCC 12,633) in alginate spheres supplemented with inorganic perlite. Regarding dry formulations, drying processes (lyophilization, air, desiccation, or spraying) are used to eliminate water to maintain a longer microbial survival time. Additional compounds that are necessary to maintain microbial survival of the carrier include the so-called “adhesives” or “protectants,” including gelatins, carboxymethyl celluloses, gum arabic, disaccharides (lactose and sucrose), and maltodextrin [120,122,162,163,164,165]. Both solid and liquid-based formulations are crucial in agriculture to nitrogen fixation, phosphorus solubilization, or nutrient mobilization, being capable of increasing soil and crop production.

The encapsulation of biofertilizers in polymeric materials also returns success similar to that usually reported for other formulations. *Rhizobium*, *Streptomyces*, *Azotobacter*, *Clostridium*, *Pseudomonas*, *Bacillus*, and *Azospirillum* have been used as biofertilizers and encapsulated in different carriers (alginate, chitosan, carrageenan, gums, gelatin, whey protein, and starch) with a wide range of application involving long exposure times in unique environmental conditions. The results indicate interesting preservation of microbial viability before being dispersed in crops, assisting in crop development [166]. Among the biopolymers listed, alginate and chitosan have been most used due to their non-toxicity, biocompatibility, and biodegradability.

Sharma et al. [71] described that the effectiveness of biofertilizers is closely related to their adequate bioformulation, without the promotion of pollutants, and with a high potential for water retention as well as simple biodegradation. FMAs are used as biofertilizers due to their ability to promote plant growth and release nutrients and absorbed water, in addition to providing resistance to soil-borne diseases. This process can be performed through FMA propagules in the form of spores or mycelia, reinforcing the importance of selecting a good carrier for adequate survival, colonization, and plant germination to occur. Formulations of the fungus *Rhizophagus clarus* in soybean (*Glycine max* L.) and corn (*Zea mays* L.) seeds, inoculated in carriers such as peat (for soybeans) and vermiculite (for corn), presented interesting results in plant development.

The application of biofertilizers requires additional precautions, preferably not storing the microbiological preparation overnight and not promptly exposing it to solar radiation, where it is recommended to store it at temperatures of 0 to 35 °C. The process of applying biofertilizers to (sterilizable) seeds requires a rehydration step with water or brown sugar, forming a homogenized paste that should be naturally sown in the field. In the case of root immersion, the biofertilizer is rehydrated with water, where the roots immersed in a diluted suspension for a certain time can then be transplanted. During application to the soil, the rehydrated biofertilizer is then sprayed before the sowing process, where the application of biofertilizers in liquid formulations dismisses the rehydration step. Therefore, thorough quality control of these bioinputs is necessary to maintain the useful life of the bioproduct for 2 to 3 months (or more with the addition of additives). It does not lose its efficiency and results in the need to supplement with chemical fertilizers [162]. A summary of registered products in Brazil is presented in Table 2.

### 9.3. Brief Survey of Patents

Bravo et al. [164], aiming for sustainable agriculture and biological control, utilized bacterial strains of the genus *Pseudomonas* sp. AMCR2b (Access Code RGM 3107) and AMTR8 (Access Code RGM 3108), isolated from the “wild flora of the Andes Mountains in the Valparaíso region”. These strains are psychrotolerant and psychrophilic, phosphate solubilizers, IAA producers, and exhibit ACC deaminase activity. They promote plant growth under cold and freezing stress by counteracting ice-nucleating bacterial phytopathogens such as *Pseudomonas syringae* and *Pectobacterium carotovorum*. Application in crops was carried out by seed immersion and spraying on roots or leaves, tested in laboratory trials with tomatoes and field trials with avocados and grapevines.

Ling et al. [165] developed a bioproduct using the endophytic fungus *Glutinomyces* sp. 32R-20, deposited at the “General Center of Microbiology of the China Microbial Culture Collection Management Committee”. This bioproduct promotes plant growth and was applied to the roots of Chinese cabbage, sugarcane, and *Dendrobium officinale*. Results highlighted a dry weight increase of 11.5 mg, larger leaf size, more tender stems, and increased cabbage diameter; greater leaf height and length in sugarcane; and weight increases from 71.31 to 122.89% in seedlings, along with significant enhancements in stem, tiller, biomass, and polysaccharide content in *D. officinale*, all compared to the control.

Xiying et al. [166] focused on preserving the black stem of *Physalis alkekengi* and *Physalis pubescens* by adding *Penicillium oxalicum* to a fermented medium with *Trichoderma viride* for biological control. These microbial agents, naturally occurring, allow for easy operation, efficient microorganism growth in co-culture or isolation, and increased root activity, characterizing it as a biocontrol product for plants.

Chung et al. [167] supplied a combination of bacteria from the genus *Pantoea* sp. NN08200, deposited under CGMCC No. 5438, which are endophytic, nitrogen-fixing, and resistant to phytopathogenic microorganisms. These bacteria were obtained from “sugarcane in Guangxi, the largest sucrose production area in China”. The inoculation of the combination into sugarcane increased nitrogen content by 41.5% and nitrogen fixation efficiency by 12.85%. In maize, nitrogen content increased by 27% after combined inoculation, and the combined strain inhibited 78.4% of the phytopathogen *Rhizoctonia solani*.

## 10. Synthetic Biology and CRISPR Editing Microorganisms

Synthetic Biology (SynBio) consists of deliberate alterations to achieve desired functions or the creation of specific components that, through transcription and translation, enable organisms to function as expected under multiple stress conditions in biological systems. To accomplish this, the combination of principles from biology, chemistry, physics, and computer science drives these modifications. Another technique that is part of SynBio is Artificial Intelligence (AI), as it incorporates various genetic model algorithms that contribute to faster, more efficient, and more precise improvements, with neural networks and support vector machines being two prominent models. Furthermore, gene editing mediated by CRISPR is a genetic engineering tool that enhances crop yield and tolerance [168]. Among the available tools in SynBio, the CRISPR-Cas system enables more precise genetic editing, which will be discussed below.

The set of clustered regularly interspaced short palindromic repeats (CRISPR) associated with a protein (Cas) (CRISPR-Cas) recognizes and cleaves exogenous viruses and plasmid DNA in bacteria, forming the acquired immune system of prokaryotes. CRISPR-Cas is divided into two subtypes: Class I includes an effector protein with a specific function in the CRISPR system, while Class II has an effector protein with multiple functions within the CRISPR system. Subtype II corresponds to CRISPR-Cas9 [169] and CRISPR-Cpf1 (also known as CRISPR-Cas12a). These two gene-editing systems have been applied to filamentous fungi, with studies showing that Cpf1 was more efficient in *Aspergillus niger*, whereas Cas9 was more effective in *Fusarium venenatum*. Single-gene editing involves small and large insertions/deletions (indels) and substitutions. To increase cellulase production through *Rasamsonia emersonii*, Cas9 was used to edit small indels, targeting the ACE1 gene and leading to the deletion of 1–2 bp of the Protospacer Adjacent Motif (PAM) sequence, rendering ACE1 non-functional. In large indels, the CRISPR-Cas system introduces two double-strand breaks (DSBs) in DNA, which are repaired through non-homologous end joining (NHEJ), leading to chromosomal fragment deletions. Two single-guide RNAs (sgRNAs) were designed for each gene in *Trichoderma harzianum* T21, resulting in the suppression of Thpyr4 (100%) and Thpks1 (89.1%). Gene substitutions involve using exogenous donor templates in high-fidelity homologous recombination (HR) repair [170].

Beyond genetic editing applied to microorganisms, as mentioned earlier, synthetic metabolic engineering driven by CRISPR and other approaches has been explored to enhance food crops. Using synthetic metabolic engineering for the biofortification of staple food crops involves transferring the CrtB and CrtI genes derived from bacteria into wheat grains, increasing carotenoid content. In maize seeds, the overexpression of phytase genes from *Aspergillus niger* enabled the development of transgenic maize containing phytase. Additionally, microorganisms can be engineered with nitrogen-fixing genes for cereal crops. SynBio can also establish a symbiotic module between fungal cultures for phosphorus acquisition in plants and biosensors (through self-monitoring, adaptive, and responsive technology—SMART) to detect fungal, bacterial, or viral infections in plants, as well as plant hormones and abiotic stress [171].

The advancement of metabolic engineering in SynBio has been enhanced by the use of artificial intelligence and machine learning, allowing for more efficient modeling and prediction of biological processes. Consequently, the SynBio programming process spans multiple areas to develop new therapies, manufacturing solutions, and environmental problem-solving strategies. A model capable of capturing non-linear biological data interactions is machine learning (ML). The ML approach has demonstrated significance in the development of AlphaFold (for protein structure predictions), BioAutoMated (for genetic sequence accessibility), Evo (a large-scale dataset model of prokaryotic genome sequences), the Automated Recommendation Tool (ART) (for engineering pathway learning tests to improve bioproduction), and a library containing over 1900 chromatin regulator (CR) pairs and their impact on transcriptional activity. However, ML has limitations when used alone, and a proposed approach for SynBio integration is hybrid models that combine ML-derived biological relationship data with adjustable physical parameters, requiring further research for better evaluation [171]. With ML integration into SynBio, significant industrial impacts are expected, accelerating innovations that drive strategic sectors such as healthcare, agriculture, and industrial biotechnology.

SynBio is an innovative vehicle for various industrial markets, including healthcare, agriculture, food, energy, and green materials. It contributes to reducing by-products, unwanted residues, process steps, and energy consumption. This has economic implications, with a current global market estimate of $12 billion across production and industrial sectors. By 2040, the market is expected to reach $767 billion, with annual growth rates between 22% and 32%. The market is driven by customer needs, and SynBio development identifies new opportunities [172].

## 11. Conclusions

The plant’s defense system initially consists of physical and biochemical barriers, with its elicitors (PAMPs/MAMPs and DAMPs) and effectors identified by receptors (PRR) triggering signaling and defense cascades (PTI or ETI), whether induced (ISR) or acquired (SAR). In addition to the harm caused by phytochemicals (eroded soil, suffering of soil microbiota, environmental contamination, and resistance of genotypes), phytopathogens have adapted to penetrate, harbor, and infect vegetable cells, requiring the plant an evolution aiming to alleviate such issues.

Biocontrol techniques using endophytic organisms, rhizosphere, and bioinputs have presented interesting effectiveness, whether with the use of a single microorganism or in combinations. The plant lacks biological controls and growth stimulation, to prevent infections from leaves and roots to maintain its homeostasis, which still are few studied due to their complexity. However, the nutritional availability promoted by bioinputs, allowing the growth and development of plants, reduces the management of chemical fertilizers, minimizing the environmental entropy. Understanding the functioning of the plant rhizosphere will likely return a better knowledge of how plant resistance responds to biotic and abiotic stresses, resulting in better yield results for crops.

Moreover, sequencing techniques using computational tools have demonstrated efficiency in reading the microbiome and its modifications for crop improvement. SynBio is characterized by the enhancement and increase of bioproduction through genetic engineering, redesigning innovative biological tools, utilizing genomic combinations, investigating libraries, and predicting functionalities. The CRISPR tool enables microbial gene editing, employing the Cas protein, which can be optimized and lead to enhanced targeting. Commercialization is challenging, and hybrid models may be more efficient, robust, and offer better interpretation. With further refinement, it is expected that SynBio integration will enable more effective outcomes, and public recognition, and contribute to agriculture and the economy. Further research is anticipated, with more genomes being decoded and the technology showing promise.

The introduction of biological approaches in sustainable agriculture represents a promising perspective for reducing dependence on chemical inputs and mitigating adverse environmental impacts. The use of beneficial microorganisms, whether for biocontrol, bio-stimulation, or biofertilization, paves the way for more resilient and regenerative agricultural practices. Combined with advancements in biotechnology and genetic engineering, such as genome editing and computational modeling of the soil microbiota, future innovations are expected to provide more efficient, sustainable, and economically viable agricultural management, significantly contributing to global food security.

## Figures and Tables

**Table 1:** Examples of producers of plant growth promoters and tested organisms with their action.

Compound	Microorganisms or Compound Producer	Tested Organisms	Remarks	Reference
IAA	Isolates of *Trichoderma* from Buenos Aires Province, in the Pampa region, Argentina	Tomato (*Solanum lycopersicum* var. platense)	High IAA production without tryptophan addition	[87]
	Isolates of *Trichoderma* spp. from Embrapa’s culture collection	‘Super Marmande’ tomato (Isla Sementes, Brazil)	IAA Production for plant growth promotion with and without the presence of L-tryptophan	[88]
	Isolates of *Bacillus* and *Pseudomonas* species from rhizosphere soil in Ethiopia	Rhizosphere of chickpea (*Cicer arietinum* L.)	High levels of IAA production using sucrose and tryptone as carbon and nitrogen sources, respectively	[89]
	*Pseudomonas*, *Serratia*, and *Stenotrophomonas* strains isolated from nodules of *Acacia mearnsii* in Paraná, Brazil	Greenhouse test with wheat	Plant growth parameters after inoculation and co-inoculation of isolated strains	[90]
	Isolates of *Diaporthe terebinthifolli* GG3F6 form India	*Glycyrrhiza glabra*	Evaluation of plant growth and IAA after inoculation of GG3F6 on *Glycyrrhiza glabra*	[91]
Phytohormones	Jasmonic acid	Microalgae (*Isochrysis zhanjiangensis*)	Physiological activities of *Isochrysis zhanjiangensis* are related to temperature variations; both wild strains and mutagenic strains showed an increase in the phytohormone IAA when incubated at 35 °C, with a more significant value in the mutagenic strain; when an inhibitor of jasmonic acid (JA, ibuprofen) was added to the wild strains, a delay in the concentration of JA and ABA occurred, allowing for growth	[92]
	Salicylic acid	Cucumber (*Cucumis sativus* cv. ‘Hangang dadagi’)	Cucumber samples susceptible to cold damage were treated with and without cooling, and salicylic acid levels increased during cold stress, along with some signaling genes (ethylene, IAA, and JA); pre-storage at 10 °C before 5 °C promoted the elimination of ROS, antioxidant accumulation, alleviating cellular damage, and causing changes in gene expression	[93]
	Ethylene	*Arabidopsis thaliana*	Phytohormones such as salicylic acid, jasmonic acid, and indole-3-acetic acid were reduced under arsenic stress, with ethylene highlighted as the precursor responsible for the reduction of arsenic accumulation in *A. thaliana* (modified), triggering signaling cascades, regulation of cellular activities, and stress resistance when compared to control seedlings	[94]
	Ethylene, gibberellic acid, and methyl jasmonate	Cassava (*Manihot esculenta* Crants)	Exogenous phytohormones are significant in reducing the rapid post-harvest physiological deterioration in cassava roots, stimulating the production of antioxidants that mitigate the accumulation of hydrogen peroxide	[95]
	Salicylic acid, ethylene, abscisic acid, and jasmonic acid	Apple (*Malus domestica* Borkh cv Fuji)	Wounds on apples induce the synthesis of phytohormones, starting with the synthesis and transduction of salicylic acid, followed by abscisic acid (ABA) and ethylene (ET); furthermore, ABA is involved in the synthesis of jasmonic acid, as is ET	[96]
Nitrogen fixation	Rhizobial inoculant: three strains of *Bradyrhizobium elkanii*	Soybean (*Glycine max* L.)	Evaluation of the effectiveness of rhizobial inoculation in soybean crops, varying the soil type between limestone and acidic soils; the results showed a stronger correlation with limestone soil, attributing the influence of slightly alkaline pH to higher nodulation, while low phosphorus concentration reduced nodulation	[97]
	Arbuscular mycorrhizal fungus: *Funneliformis mosseae* (or *Glomus mosseae*)	Tomato (*Solanum lycopersicum* var. platense)	Mycorrhizal fungi provide plant roots with the ability to absorb reasonable amounts of methionine, cysteine, and inorganic nitrogen (after mineralization), during competition with other microorganisms present in the soil	[98]
	Arbuscular mycorrhizal fungus: *Rhizophagus intraradices*	Wheat (*Triticum aestivum*)	Colonization of arbuscular mycorrhizal fungi in host plants enhances the absorption and transport of nitrogen in both organic and inorganic forms due to the interaction of the mycorrhizal hyphae within the mycorrhizosphere; this resulted in increased nitrate acquisition in wheat plants grown in soils contaminated with arsenic	[99]
	Mixed inoculum with six species of arbuscular mycorrhizal fungi (AMF): *Funneliformis geosporum*, *F. mosseae*, *Glomus versiforme*, *Acaulospora scrobiculata*, *Rhizophagus intraradices*, and *Gigaspora margarita*	Wheat seed (*Triticum aestivum* L. var. Yangfumai 4) with the aid of earthworms present in the soil (*Eisenia fetida* epigeic and *Metaphire guillelmi* endogeic), added after 69 days of sowing	Low carbon (C) levels decrease NH_4_^+^ and increase NO_3_^−^; however, excessive C can suppress denitrification or increase the proliferation of AMF hyphae; earthworms affect nitrogen transformation in the soil by AMF through their hyphae in relation to different feeding points with crop residues and the dissemination of their spores; the depth, soil porosity, and aggregate stability where the crop residues are located affect the performance of the earthworms	[100]
	Mycorrhizal symbiosis	Soybean (*Glycine max* (L.) Merr.)	Environments contaminated with polypropylene reduced water consumption efficiency, altering the physiological functioning of plants; additionally, the symbiosis of mycorrhizal fungi did not contribute to improving the situation, as in favorable environments, plants may not benefit if the mycorrhiza is not properly managed; in this case, the mycorrhiza competes with the plant for water and nutrients, hindering plant growth	[101]
Phosphate	*Bacillus subtilis* and *Methylobacterium organophyllum*	Rhizobacteria in Wheat Crops	Inoculation of tricalcium with *Bacillus subtilis* and *Microbacterium organophyllum* led to increased phosphorus solubilization, absorption, and improved phosphorus use efficiency	[102]
	*Enterobacter* sp.	Green gram (*Vigna radiata*)	Phosphate-solubilizing bacteria isolated from the Bengal region, eastern India, showed promising results and demonstrated greater efficiency in liquid bioformulations when polymer additives were added, compared to solid carriers	[103]
	*Aspergillus flavus*	-	The fungal strain was able to recover phosphorus from various solid wastes such as biochar and ash-based sludge, increasing the solubilization of insoluble phosphate; it shows potential as an agricultural biofertilizer	[104]
	Endophytics *Burkholderia vietnamiensis* and *Paraburkholderia kururiensis* isolated from *Oryza sativa* subsp. *Indica xiangzaoxian*	*Brachypodyum distachyon* and *Arabiopsis thaliana*	*Paraburkholderia kururiensis* showed endophytic potential and plant growth promotion capabilities; while *Burkholderia vietnamiensis* is proficient in nitrogen fixation and phosphate solubilization, this study demonstrated functional divergence	[105]
	Isolates of Barley (*Hordeum vulgare* L.)	-	*Exophiala equina* and *Curvularia arcana* strains solubilize Ca(PO_4_)_2_ and AlPO_4_; *Clonostachys solani* f. *nigrovirens* solubilizes Ca(PO_4_)_2_ and FePO_4_, while *Clonostachys rosea* f. *catenulata* and *Microdochium* sp. only solubilize Ca(PO_4_)_2_; these are candidates for use as bioinoculants, with strains *C. rosea* f. *catenulata*, *Microdochium* sp., and *C. arcana* favored due to their rapid growth, and *E. equina* and *C. solani* f. *nigrovirens* showing slower growth rates; all of them exhibit a high relative solubilization efficiency index	[106]
Siderophores	Enteroquelina (*Koasakonia radicintans*)	-	Higher enterochelin production by *Klebsiella radicincitans* using lactose as a carbon source	[107]
	*Trichoderms* spp., *Fusarium* spp., and *Aspergillus nidulans*	-	Production of different types of siderophores: coprogen, by *Trichoderma fusigenum*, by *Fusarium*, and ferricrocin and ferrihydrin by *Aspergillus*	[82]
	Enterobactin siderophore (*Escherichia coli*); Methanobactin (*Metilosinus tricosporium*)	-	Cancer therapy: reveals antitumor activity in tumor cell lines associated with monocytes; Metal chelation therapy: chelation of copper that prevents liver failure in mice with Wilson’s disease	[108]
	Endophytic fungi *Penicillium glabrumi* and *Aspergillus niger*	*Jasminum sambac*, *Camellia sinensis*, and *Ocimum basilicum* in the Riad region, Saudi Arabia	Characterize (IAA and siderophore) the endophytic microorganism of medicinal host plants and quantify compounds that promote plant growth	[109]
	Pyoverdine siderophore (*Pseudomonas* sp.)	*Arabidppsis thaliana*	Induction of plant development associated with the chelating ability of siderophores	[110]
Volatile compounds	Alkane and 3-pentanol	*Arabidopsis thaliana* and field plants	Alkane tridecane produced by *Paenibacillus polymyxa* induces the pathogenesis-related (PR) response in *A. thaliana* and provides defense against *Pseudomonas syringae*; seed treatment with 3-pentanol produced by *Bacillus amyloliquefaciens* provides defense against *Xanthomonas axonopodis* pv. *vesicatoria*, inducing phytohormones (salicylic acid, jasmonic acid, and ethylene)	[111]
	3-Octanone and hexadecane	*Nicotiana benthamiana*	Volatile compounds from the oomycete *Pythium oligandrum* in *N. benthamiana* seedlings led to an increase in the expression of growth-related genes	[112]
	Acetoin (3-Hydroxybutan-2-one)	*Arabidopsis thaliana*	The volatile organic compound acetoin produced by *Bacillus mojavensis* induces systemic resistance in *A. thaliana*; additionally, the bacterium exhibits antagonistic activity against *Fusarium verticillioides*, *Fusarium graminearum*, and *Rhizoctonia solani*	[113]
	3-Methylbutyl, 3-octanone, nonanal, β-farnesene, β-bisabolene, β-sesquifelandrene, 1-octen-3-ol, 3-octanone, 1-octen-3-ol, 3-octanone, β-sesquifelandrene, β-bisabolene, γ-muurolene, and acoradiene	Alface (*Lactuca sativa* L.)	Volatile organic compounds emitted by *Trichoderma azevedoi* promote lettuce growth and increase chlorophyll and carotenoid content; it also shows potential during sporulation to inhibit *Sclerotinia sclerotiorum*	[114]
	Auxin and ethylene	*Arabidopsis thaliana*	Volatile organic compounds produced by *Papiliotrema flavescens*, applied to *A. thaliana*, showed greater effects on the roots than on the shoot; these compounds affect hormonal signaling, plant development (auxin and ethylene), and defense responses	[115]

**Table 2:** Formulated products from the Ministry of Agriculture, Livestock, and Supply (Brazil)—General Coordination of Pesticides and Related Products/DFIA/SDA [163].

Brand Name	Registration Title **	Registration Number	Active Ingredient (Chemical Group)	Treatment
* Biogalloi	Morsoletto Controle biológico Ltda.	6621	*Trichoderma galloi* (biological)	Foliar
Bioturim	Total Biotecnologia Industria e Comercio S/A—Curitiba/PR	10,224	*Bacillus thuringiensis*, isolates CCTB22, CCTB23 and CCTB25 (biological)	Foliar
BTP 500-21A	Total Biotecnologia Industria e Comercio S/A—Curitiba/PR	10,124	*Bacillus subtilis*, isolates CBMAI 1680 and CNPSo 2657 (biological)	Foliar
* Thereos	Companhia Nitro Química Brasileira—Sertãozinho/SP	24,123	*Telenomus podisi* (biological)	Foliar
* Trichobiogramma	Insecta Bio Indústria e Comércio Ltda.—Campo Verde/MT	37,119	*Trichogramma pretiosum* (biological)	Foliar
Acera	Ballagro Agro Tecnologia Ltda.—Bom Jesus dos Perdões/SP	14,320	*Bacillus thuringiensis* (microbiological)	Foliar
Aevo	Total Biotecnologia Industria e Comercio S/A—Curitiba/PR	15,522	*Pseudomonas chlororaphis*, isolate CCTB19 (microbiological) + *Pseudomonas fluorescens*, isolate CCTB03 (microbiological)	Foliar
Agree	Bio Controle—Métodos de Controle de Pragas Ltda.	6095	*Bacillus thuringiensis* (microbiological)	Foliar
AgTecmmon	Massen Produtos Biológicos S.A—Indaiatuba/SP	24,820	*Bacillus amyloquefaciens*, isolate CPQBA 040-11DRM 01 (microbiological) + *Bacillus amyloquefaciens*, isolate CPQBA 040-11DRM 04 (microbiological)	Foliar
Álaabo; Isatrix; Isashock	Agrobiológica Sustentabilidade S.A—Filial	40,719	*Paecilomyces fumosoroseus* (microbiological)	Foliar
* Aleris	Alfa Agotec Produtos Agrícolas Ltda.	27,524	*Beauveria bassiana*, isolate IBCB 66 (microbiological)	Foliar
Aptur-PF	Agrobiológica Sustentabilidade S.A.—Filial	24,618	*Paecilomyces fumosoroseus* (microbiological)	Foliar
* Aradya	Genica Inovação Biotecnológica S.A.—Planta2/Piracicaba/SP	1720	*Metarhirium anisopliae*, isolate IBCB 425 (microbiological)	Foliar
Armigen	Agbitech Controles Biológicos Ltda.	7815	VPN-HzSNPV (microbiological)	Foliar
* Atrevido	Koppert do Brasil Holding S.A—Piracicaba/SP	32,217	*Beauveria bassiana*, isolate IBCB 66 (microbiological)	Foliar
Atroverde 77; T-77	Andermatt do Brasil Soluções Biológicas Ltda.—ME	5423	*Trichoderma atroviride* (microbiological)	Foliar
Auin	Produtos Agrícolas S.A—Indaiatuba/SP	14,324	*Beauveria bassiana*, isolate IBCB 66 (microbiological)	Foliar
BAc Control Max EC	Vectorcontrol Industria e Comercio de Produtos Agropecuários Ltda.—Vinhedo/SP	30,518	*Bacillus thuringiensis* (microbiological)	Foliar
* *Bacillus thuringiensis* Bom Futuro	Bom Futuro Agrícola Ltda.—Campo Verde/MT	5022	*Bacillus thuringiensis* var. kurstaki, isolate HD-1 (S1450) (microbiological)	Foliar
* Bacmix BTKSC	Cooperativa Mista de Desenvolvimento do Agronegócio/COMDEAGRO—Primavera do Leste/MT	2420	*Bacillus thuringiensis* var. kurstaki, isolado HD-1 (S1450) (microbiological)	Foliar
* Bactel; DuoBac Meta; Easy Amylo; Baculomip-SF; Spinix	Dillon Biotecnologia Ltda.	22,620	*Bacillus amyloquefaciens* (microbiological)	Foliar
* Batuk; Kubera	Solatus Biotecnologia e Insumos Ltda.—Jardinopolis/SP	28,622	*Beauveria bassiana*, isolate IBCB 66 (microbiological)	Foliar
BB-Protec, Beauvisan	Andermatt do Brasil Soluções Biológicas Ltda.—ME	9723	*Beauveria bassiana* (microbiological)	Foliar
* Beauve 100; Titanium; Bove; BioCAZ Beauveria	Oligos Biotecnologia Fabricação de Defensivos Agrícolas Ltda.	7622	*Beauveria bassiana*, isolate IBCB 66 (microbiological)	Foliar
* BeauveOuro	Valeouro Biotec Ltda.—Uberaba/MG	30,522	*Beauveria bassiana*, isolate IBCB 66 (microbiological)	Foliar
* Beauveria Bom Futuro	Bom Futuro Agrícola Ltda.—Campo Verde/MT	19,722	*Beauveria bassiana*, isolate IBCB 66 (microbiological)	Foliar
* Beauveria SR	Fabricação de DefensivosAgrícolas Ltda.	27,020	*Beauveria bassiana*, isolate IBCB 66 (microbiological)	Foliar
Betk-03	Bio Insumos Nativa do Brasil Ltda.—Hortolândia/SP	7123	*Bacillus thuringiensis*, strain N1 + *Bacillus thuringiensis*, strain N3 + *Bacillus thuringiensis*, strain N3 (microbiological)	Foliar
* Bio Centules	Bionat Soluções Biologicas Ltda.	21,222	*Beauveria bassiana*, isolate IBCB 66 (microbiological)	Foliar
* Bio Green; Marecjal—Bioagreen; Trichomais PM; Green Bio	Solubio Tecnologias Agrícolas S.A.—Jataí/GO	28,422	*Trichoderma harzianum*, isolate IB 19/17 (microbiological)	Foliar
* Bio Phygga	Bionat Soluções Biologicas Ltda.	12,422	*Metarhizium anisopliae*, isolate IBCB425 (microbiological)	Foliar
* Bioatena	Total Biotecnologia Industria e Comercio S/A—Curitiba/PR	25,121	*Metarhizium anisopliae*, isolate IBCB425 (microbiological)	Foliar
Bioatria	Bionat Soluções Biologicas Ltda.	25,522	*Trichoderma afroharzianum*, strain CEN287 (microbiological)	Foliar
Amanzi	Agrobiológica Sustentabilidade S.A.—Filial	21,721	*Bacillus amyloquefaciens*, (microbiological)	Foliar
Biodiatraea	Morsoletto Controle Biológico Ltda.	7522	*Trichospilus diatraeae* (biological)	Foliar
Biopalmis	Morsoletto Controle Biológico Ltda.	6422	*Palmistichus elaeises* (biological)	Foliar
Biopodisi	Morsoletto Controle Biológico Ltda.	822	*Telenomus podisi* (biological)	Foliar
Bioprecioso	Morsoletto Controle Biológico Ltda.	20,522	*Trichogramma pretiosum* (biological)	Foliar
Catolaccus Ampia	Associação Mineira dos Produtores de Algodão—AMIPA	15,822	*Catolaccus grandis* (biological)	Foliar
Defender	CL Empreendimentos Biológicos Ltda.	30,921	*Telenomus podisi* (biological)	Foliar
Podisibug	Koppert do Brasil Macrobiologicos Ltda.—Charqueada/SP	43,919	*Telenomus podisi* (biological)	Foliar
Bioagro Raiz	Bioma Indústria Comércio e Distribuição Ltda.—Fazenda Rio Grande/PR	226,223	*Bacillus valezensis*, isolate CCT7943 (biological)	Seeds
Bioagro Solo	Simbiose Indústria e Comércio de Fertilizantes e Insumos Microbiológicos Ltda.	37,819	*Trichoderma harzianum* (microbiological)	Seeds
Biolucro	Total Biotecnologia Industria e Comércio S/A—Curitiba/PR	23,123	*Bacillus circulans*, isolate CCT0026 (biological) + *Bacillus licheniformis*, isolate CCTB07 (biological) + *Bacillus subtilis*, isolate CCTB04 (biological) + *Paenibacillus azotofixans*, isolate CCT4719 (biological)	Seeds
Bioma—B. Bv 10	Bioma Indústria Comércio e Distribuição Ltda.—Fazenda Rio Grande/PR	22,523	*Bacillus velezensis*, isolate CCT7944 (biological)	Seeds
Biomagno; Bioharmer	Total Biotecnologia Indústria e Comércio S/A—Curitiba/PR	11,422	*Bacillus amyloquefaciens*, isolate CNPSo3202 (microbiological) + *Bacillus thuringiensis*, isolate CNPSo3915 (microbiological) + *Bacillus velezensis*, isolate IBLF1278 (microbiological)	Seeds
* Biotricho	Biomip Agentes Biológicos Ltda.	11,522	*Trichoderma harzianum*, isolate IBLF1278 (microbiological) + *Trichoderma harzianum* isolate IBLF1282 (microbiological) + *Trichoderma viride*, isolate IBLF1275 (microbiological) + *Trichoderma viride*, isolate IBLF 1276 (microbiological)	Seeds
Biotrinsic D451 FP; Biotrinsic Hamatum; Biotrinsic Fitocontrol; Indigo 451; Indigo Hamatum	Indigo Brazil Agricultura Ltda.	2224	*Trichoderma hamatum*, strain SYM37537 (biological)	Seeds
Boneville	Koppert do Brasil Holding S.A.—Piracicapa/SP	11,720	*Bacillus amyloliquefaciens* (microbiological)	Seeds
BTP 007-19	Total Biotecnologia Industria e Comercio S/A—Curitiba/PR	31,022	*Bacillus velezensis*, isolate CNPSo3602 (microbiological)	Seeds
BTP 177-21	Total Biotecnologia Industria e Comercio S/A—Curitiba/PR	31,423	*Bacillus firmus* CCT0227 (biological)	Seeds
BTP 177-21A	Total Biotecnologia Industria e Comercio S/A—Curitiba/PR	36,024	*Bacillus firmus* CCT0227 (biological)	Seeds
Certano	Syngenta Proteção de Cultivos Ltda.—São Paulo/SP	3121	*Bacillus velezensis*, isolate CNPSo3602 (microbiological)	Seeds
Chevelle	Koppert do Brasil Holding S. A.—Piracicaba/SP	11,820	*Bacillus amyloliquefaciens* (microbiological)	Seeds
Clariva PN	Syngenta Proteção de Cultivos Ltda.—São Paulo/SP	16,917	*Pasteuria nishizawae* (microbiological)	Seeds
Clariva PN BR	Syngenta Proteção de Cultivos Ltda.—São Paulo/SP	14,418	*Pasteuria nishizawae* (microbiological)	Seeds
Duonasty	Agrobiológica Sustentabilidade S. A—Filial	33,324	*Bauveria bassiana* ASN 053 (biological) + *Metarhizium anisopliae* ASN054 (biological)	Seeds
Faciens Protection	Simbiose Indústria e Comércio de Fertilizantes e Insumos Microbiológicos Ltda.	22,018	*Bacillus amyloliquefaciens* (microbiological)	Seeds
Inlayon	Simbiose Indústria e Comércio de Fertilizantes e Insumos Microbiológicos Ltda.	8318	*Bacillus amyloliquefaciens* (microbiological)	Seeds
Inlayon Eco	Simbiose Indústria e Comércio de Fertilizantes e Insumos Microbiológicos Ltda.	35,021	*Bacillus amyloliquefaciens* (microbiological)	Seeds
Lalnix Resist	Lallemand Soluções Biológicas Ltda.—Piracicaba/SP	20,518	*Trichoderma endophyticum*, isolate IBCB 56/12 (microbiological)	Seeds
* Lalstop Organic DS	Lallemand Soluções Biológicas Ltda.—Piracicaba/SP	10,322	*Trichoderma asperellum*, isolate URM-5911 (microbiological)	Seeds
Loyalty Bio; Trunemco; Vinemco	Prophyto Comercio e Serviõs Ltda.—São Paulo/SP	43,019	*Bacillus amyloliquefaciens* (microbiological)	Seeds
* Nem Phoco	Tecnologia Ltda.—Primavera do Leste/MT	8223	*Trichoderma harzianum*, isolate IBLF1278 (microbiological) + *Trichoderma harzianum*, isolate IBLF1282 (microbiological) + *Trichoderma harzianum*, isolate IBLF1275 (microbiological) + *Trichoderma harzianum*, isolate IBLF1276 (microbiological)	Seeds
Nemacontrol	Simbiose Indústria e Comércio de Fertilizantes e Insumos Microbiológicos Ltda.	12,016	*Bacillus amyloliquefaciens* (microbiological)	Seeds
Nemat Stellus	Ballagro Agro Tecnologia Ltda.—Bom Jesus dos Perdões/SP	6224	*Paecilomyces lilacinus*, strain URM7661 (biological) + *Pochonia chalmyloliquefaciens*, strain URM8121 (biological)	Seeds
Nemax	Bioma Indsústria Comércio e Distribuição Ltda.—Fazenda Rio Grande/PR	22,423	*Bacillus amyloliquefaciens*, isolate CBMAI2356 (microbiological)	Seeds
Nimaxxa	CHR Hansen Indústria e Comercio Ltda.	24,222	*Bacillus paralicheniformis*, isolate CH0273 (microbiological) + *Bacillus paralicheniformis*, isolate CH2970 (microbiological) + *Bacillus subtillis*, isolaye CH4000 (microbiological)	Seeds
Oleaje Prime	Basf S. A.—São Paulo	32,817	*Bacillus firmus* (microbiological)	Seeds
* Onix OG	Lallemand Soluções Biologicas Ltda.—Piracicaba/SP	15,216	*Bacillus methylotrophicus*, isolate UFPEDA20 (microbiological)	Seeds
Presence; Fortmax	FMC Química do Brasil Ltda.—Campinas/SP	1817	*Bacillus licheniformis* (microbiological) + *Bacillus subtilis* (microbiological)	Seeds
Protege	Adama Brasil S. A.—Londrina/PR	10,822	*Bacillus amyloliquefaciens*, isolate CNPSo3202 (microbiological) + *Bacillus thuringiensis*, isolate CNPSo3915 (microbiological) + *Bacillus velezensis*, isolate CNPSo 3602 (microbiological)	Seeds
Pusher	Total Biotecnologia Industria e Comercio S/A—Curitiba/PR		*Bacillus subtilis*, isolate CCTB04 (Biological)	Seeds
Quorum	Total Biotecnologia Industria e Comercio S/A—Cuitiba/PR	19,922	*Bacillus amyloliquefaciens*, isolate CNPSo3202 (microbiological) + *Bacillus thuringiensis*, isolate CNPSo3915 (microbiological) + *Bacillus velezensis*, isolate CNPSo 3602 (microbiological)	Seeds
Rizoderma TSI; Rizoderma TSI Bio Fungicida; Rizoderma	Rizobacter do Brasil Ltda.	29,421	*Trichoderma afroharzianum*, strain Th2RI99 (microbiological)	Seeds
* Rizos	Lallemand Soluções Biológicas Ltda.—Piracicaba/SP	15,116	*Bacillus subtilis*, isolate UFPEDA764 * (microbiological)	Seeds
* Tivra	Solatus Biotecnologia e Insumos Ltda.—Jardinopolis/SP	1823	*Trichoderma harzianum*, isolate IBLF1278 (microbiological) + *Trichoderma harzianum*, isolate IBLF1282 (microbiological) + *Trichoderma viride*, isolate IBLF1275 (microbiological) + *Trichoderma viride*, isolate IBLF1276 (microbiological)	Seeds
T-Protec	Soluções Biológicas Ltda.—ME	31,623	*Trichoderma asperellum* (microbiological)	Seeds
* Trichoderma Bom Futuro	Bom Futuro Agrícola Ltda.—Campo Verde/MT	33,122	*Trichoderma harzianum*, isolate IBLF1278 (microbiological) + *Trichoderma harzianum*, isolate IBLF1282 (microbiological) + *Trichoderma viride*, isolate IBLF1275 (microbiological) + *Trichoderma viride*, isolate IBLF1276 (microbiological)	Seeds
Veraneio	Koppert do Brasil Haolding S.A.—Piracicaba/SP	11,620	*Bacillus amyloliquefaciens* (microbiological)	Seeds
Voluto	Ballagro Agro Tecnologia Ltda.—Bom Jesus dos Perdões/SP	6324	*Paecilomyces lilacinus* strain URM 7661 (biological) + *Pochonia chlamydosporia* strain URM 8121 (biological)	Seeds

* Phytosanitary product approved for use in organic farming; ** In Portuguese (as it is registered).

## Data Availability

No new data were created or analyzed in this study.

## Abbreviations

The following abbreviations are used in this manuscript:

AUX	Auxin
CKs	Cytokines
GAs	Gibberellin
ET	Ethylene
ABA	Abscisic acid
SA	Salicylic acid
JA	Jasmonic acid
PTI	Pattern-triggered immunity
MAPKs	Mitogen-activated accumulation
PAMPs/MAMPs	Non-pathogenic or pathogenic microorganisms
DAMPs	Demage occurs to their cells
PRRs	Pattern recognition receptors
LRRs	Leucine-rich extracellular repeats
RLKs	Receptor-like kinases
RLPs	Receptor-like protein
LysM	Lysin motif
EGF	Epidermal growth factor
RLCKs	Receptor-like cytoplasmic kinases
MTI	Microorganisms-triggered immunity
NHRs	Non-host pathogens
CaM	Calmodulin
ROS	Reactive oxygen species
ETS	Effector-triggered susceptibility
NLRs	Nod-like receptors
RNS	Reactive nitrogen species
NO	Nitric oxide
EE	Early endosome
TGN/EE	Trans-Golgi network
MeSA	Methyl salicylic acid
G3P	Glycerol-3-phosphate
PRs	Pathogenesis-related proteins
SAR	Systemic acquired resistance
LPS	Lipopolysacharydes
TTSS	Secreted by type-III secretion system
AMF	Arbuscular mycorrhizal fungi
PMB	Phosphorus mobilizing bacteria
QS	Quorum sensing
VOCs	Volatile organic compounds
IAA	Indole-3-acetic acid
PBRMs	Plant-beneficial rhizospheric microorganisms
PGPR	Plant growth-promoting rhizobacteria
ACC	Aminocyclopropane-1-carboxylic acid
BCAs	Biological control agents
MFs	Mycorrhizal fungi
ECTO	Ectomycorrhizae
ENDO	Endomycorrhizae
AM	Arbuscular mycorrhizae
EM	Ericoid mycorrhizae
EFF	Endophytic filamentous fungi
PGPF	Plant-growth-promoting fungi
EPGPB	Endophytic plant growth-promoting bacteria
HA	Humic acid
FA	Fulvic acid
HU	Humins
ISR	Induction of resistance, whether induced
BAX	Bcl-2-associated X
EV	Empty vector
Cu	Copper
Ni	Nickel
Zn	Zinc
SynBio	Synthetic Biology
IA	Artificial Intelligence
CRISPR	Clustered Regularly Interspaced Short Palindromic Repeats
NHEJ	Non-homologous end joinig
sgRNA	Singles-guide RNA
HR	High-fidelity homologous recombination
SMART	Self-monitoring, adaptive, and responsive technology
ML	Machine learning
AlphaFold	Access to protein structure predictions
BioAutoMated	Genetic sequence accessibility
Evo	Large-scale model of prokaryotic genome DNA sequence datasets
ART	Automated Recommendation Tool
CR	Chromatin
